# Plant Responses to Heavy Metal Stress: Molecular Mechanisms, Transcriptomic Regulation, and Multi-Omics Integration in Crop Improvement

**DOI:** 10.3390/plants15192977

**Published:** 2026-09-29

**Authors:** Zagipa Sapakhova, Dias Daurov, Ainash Daurova, Xiaoxia Luo, Zhanar Abilda, Maxat Toishimanov, Rakhim Kanat, Iskander Isgandarov, Kabyl Zhambakin, Malika Shamekova

**Affiliations:** 1 Laboratory of Breeding and Biotechnology, Institute of Plant Biology and Biotechnology, Timiryazev 45, Almaty 050040, Kazakhstan; 2 Tanir Research Laboratory, 75B Al-Farabi Avenue, Almaty 050060, Kazakhstan; 3 Faculty of Agrobiology, Kazakh National Agrarian Research University, Almaty 050010, Kazakhstan; 4 College of Life Science, Tarim University, Alar 843300, China

**Keywords:** heavy-metal stress, plant tolerance, transcriptomics, transcription factors, metal transporters, multi-omics

## Abstract

Heavy-metal contamination poses a serious threat to plant growth, productivity, and resilience, as it causes complex changes in cellular homeostasis, redox balance, element transport, and metabolism. This review summarizes current understanding of the molecular mechanisms by which plants adapt to heavy-metal stress, with a particular focus on transcriptional and omics approaches. The main detoxification and defense mechanisms are discussed, including chelation and vacuolar sequestration, antioxidant regulation, hormonal signaling, and ion transport. Particular attention is paid to the role of MAPK cascades and transcription factors in coordinating the expression of genes involved in metal transport, maintenance of redox homeostasis, and detoxification. Analysis of transcriptomic studies shows that the plant response is characterized by pronounced temporal, tissue-specific, and genotypic specificity and involves the reprogramming of genes involved in transport, antioxidant defense, secondary metabolism, and signaling pathways. At the same time, transcript levels do not always reflect the functional state of proteins and metabolic processes. Therefore, the integration of transcriptomics with genomics, epigenomics, proteomics, metabolomics, and single-cell and spatial approaches combined with functional validation is proposed as a key direction that may facilitate the identification of robust molecular markers and functionally validated genes for improving crop tolerance to heavy-metal contamination.

## 1. Introduction

Heavy-metal (HM) contamination, particularly in areas of mining activity, is one of the primary sources of abiotic stress for plants, significantly affecting their physiology, growth, and survival in contaminated environments [1]. Due to their high toxicity, persistence, and potential for bioaccumulation, metals such as cadmium (Cd), lead (Pb), mercury (Hg), molybdenum (Mo), and arsenic (As) pose a serious environmental and agricultural problem. They enter the soil primarily through industrial effluents, mining waste, and agrochemicals, degrading soil quality, reducing crop yields, and increasing the risk of food contamination [2,3,4].

Heavy metals disrupt the morphological, physiological, and biochemical processes of plants, with photosynthesis being one of the most sensitive processes. Their accumulation in plant tissues causes a decrease in CO_2_ concentration in the leaf mesophyll, disrupts stomatal function, reduces stomatal conductance, transpiration, and photosynthetic activity, and also leads to an imbalance in ion exchange, disruption of water balance, and increased formation of reactive oxygen species (ROS) [5,6,7,8,9,10,11,12]. As a result, growth is slowed, and plant biomass and yield are reduced.

In response to heavy-metal exposure, plants activate a complex of interconnected adaptive mechanisms to limit metal uptake and transport, maintain ionic and redox homeostasis, and prevent cellular damage. These processes include the regulation of metal uptake and transport; antioxidant defense; Ca^2+^/ROS and MAPK signaling; hormonal regulation; transcriptional activation of stress-response genes; metal chelation with phytochelatins, glutathione, and metallothioneins; and their compartmentation and sequestration in the cell wall and vacuoles [13]. Rhizospheric microorganisms and mycorrhizal fungi, which can alter metal bioavailability and modulate plant stress responses, further contribute to plant tolerance [14]. Modern research is aimed not only at uncovering these molecular mechanisms but also at their practical application in phytoremediation, including phytoextraction and phytostabilization [15]; the use of metal-resistant microorganisms [16]; and microalgae [17], nanotechnology [18], and molecular genetic approaches [19]. However, the effectiveness of these strategies depends on the type of metal, plant genotype, environmental conditions, and interactions among multiple stress factors, necessitating a comprehensive consideration of adaptation mechanisms and modern approaches to reducing heavy-metal toxicity.

Advances in high-throughput molecular technologies have significantly expanded our understanding of how plants respond to heavy metals. Genomics identifies genes potentially involved in metal transport and detoxification [20], while transcriptomics reveals stress-induced changes in expression and regulatory pathways [21]. However, isolated omics approaches do not capture the full complexity of the stress response, as transcript levels do not always correlate with protein content and activity, and metabolic changes can be regulated independently of transcription [20]. Therefore, integrating genomic, transcriptomic, proteomic, and metabolomic data within a systems biology framework enables a comprehensive understanding of the mechanisms underlying heavy-metal tolerance [19]. Combined with high-throughput phenotyping and QTL mapping, multi-omics approaches help identify key resistance loci and markers, supporting marker-assisted and genomic selection [19,22]. Furthermore, integrating multi-omics with CRISPR/Cas technologies and research into plant-microbe interactions creates opportunities to precisely modify resistance genes and develop effective bioinoculants [22].

This review examines the molecular and physiological mechanisms underlying plant adaptation to heavy-metal stress, with particular emphasis on transcriptional regulation and the interplay between mitogen-activated protein kinase (MAPK) signaling and transcription factors in antioxidant defense, metal transport, chelation, and vacuolar compartmentalization. Transcriptomic evidence is considered alongside genomic, epigenomic, proteomic, metabolomic, and ionomic perspectives to provide a broader understanding of the regulatory mechanisms underlying heavy-metal adaptation. Finally, the integration of multi-omics approaches with high-throughput phenotyping, CRISPR/Cas technologies, and plant–microbe interactions is highlighted as a promising future direction that may advance systems-level understanding and facilitate the identification and functional validation of molecular targets for crop breeding and enhanced resilience.

Beyond describing the molecular mechanisms underlying heavy-metal responses, this review evaluates their potential relevance for crop improvement. Particular attention is given to traits that can be translated into breeding targets, including reduced uptake and root-to-shoot translocation of toxic metals, enhanced cellular detoxification and sequestration, maintenance of essential-element homeostasis, and preservation of growth and yield under contaminated conditions. The discussion further addresses how genomic, transcriptomic, multi-omics, and functional-genomics approaches can support the identification and validation of candidate breeding targets, while distinguishing physiological tolerance from reduced metal accumulation in edible tissues and other food-safety traits.

This review was conducted as a narrative and integrative literature review. Relevant literature was identified through (i) searches of Web of Science, Scopus, and PubMed; (ii) the detailed database-specific search strategies are provided in Supplementary Table S1; (iii) consideration of peer-reviewed literature published primarily from 2010 to September 2026, with emphasis on recent studies; and (iv) inclusion of English-language publications directly relevant to plant responses to heavy-metal stress, while studies outside the scope of the review were excluded.

## 2. Major Heavy Metals and Their Effects in Plants

Heavy metals are conventionally classified as essential and non-essential elements. Essential elements, including Fe, Cu, Zn, Mn, Ni, Co, Al, and Mo, are necessary for normal plant growth and development, as they participate in chlorophyll and protein biosynthesis, electron transport, redox reactions, respiration, lignin biosynthesis, enzyme activity, and nitrogen assimilation. However, excess levels of these elements cause disruptions in cellular metabolism, oxidative stress, and growth inhibition [23,24]. Non-essential elements, such as Cd, Pb, Hg, As, and Cr, do not perform any known physiological functions and exhibit pronounced phytotoxicity, disrupting cellular homeostasis, photosynthesis, mineral nutrition, and plant productivity [25,26].

Cadmium is one of the most toxic soil contaminants and causes oxidative stress, disruption of mineral nutrition, chlorophyll degradation, membrane damage, and inhibition of plant growth [27,28]. Lead accumulates primarily in the root system and disrupts growth, photosynthesis, water, and mineral metabolism, while simultaneously stimulating the formation of ROS and damaging cellular structures [29,30,31]. Chromium disrupts photosynthesis, water and mineral metabolism, inhibits growth, and induces oxidative stress, leading to damage to membranes and DNA [32]. Arsenic disrupts germination, nutrient uptake, and photosynthetic activity, while simultaneously increasing ROS production and oxidative damage to cells [33]. Mercury is highly phytotoxic and causes disturbances in growth, photosynthesis, and mineral nutrition, as well as oxidative stress, lipid peroxidation, and DNA damage [34].

Copper and Zinc are essential micronutrients required for the functioning of the photosynthetic and enzymatic systems; however, excess levels of these elements cause oxidative stress, disrupt nutrient uptake and photosynthesis, and reduce plant growth [35,36,37,38,39,40,41,42,43,44,45,46]. Iron plays an essential role in chlorophyll synthesis, electron transport, and redox reactions. Manganese is required for photosynthesis and water splitting in photosystem II, and nitrogen metabolism. Nickel is essential for urease activity and nitrogen metabolism. Cobalt contributes to nitrogen fixation and nodule development, particularly in legumes. Molybdenum is required for nitrate reduction, nitrogen assimilation, and biological nitrogen fixation [23,35]. Although molybdenum is an essential micronutrient, excessive Mo accumulation can cause toxic effects in plants and microalgae. In higher plants, Mo toxicity is associated with impaired growth and chlorosis, with its severity depending strongly on soil properties, Mo bioavailability, and plant species [47]. In *Chlamydomonas reinhardtii*, high molybdate concentrations can also inhibit growth, highlighting the importance of tightly regulated Mo uptake and intracellular homeostasis [48].

Aluminum is typically considered separately from typical heavy metals as one of the main factors contributing to the toxicity of acidic soils. At low pH, its solubility and bioavailability increase, leading primarily to the inhibition of root growth and disruption of water and mineral nutrient uptake [49].

Thus, despite differences in toxicity and mechanisms of action among individual metals, their effects are often accompanied by oxidative stress, disruption of ion homeostasis, photosynthesis, and growth. These general and specific responses form the basis of the plant’s molecular response, including changes in gene expression, regulation of ion transport, chelation, and activation of antioxidant defenses.

## 3. Plant Defense Mechanisms

Plant tolerance to heavy metals is mediated by an interconnected network of processes that operates from the initial entry and perception of metal ions to their detoxification and intracellular sequestration. In this context, tolerance refers to the ability of plants to maintain growth and physiological function under metal exposure and should be distinguished from metal exclusion, accumulation, and food-safety traits. Exclusion involves restricting metal uptake or translocation, whereas accumulation refers to the retention or concentration of metals within plant tissues. Detoxification includes processes such as chelation, sequestration, compartmentalization, and redox regulation that reduce the cellular toxicity of accumulated metals. Importantly, physiological tolerance does not necessarily result in low metal concentrations in edible tissues; a plant may tolerate and detoxify substantial metal loads while still accumulating metals in aboveground or edible organs. Conversely, reduced metal accumulation in edible tissues represents a distinct food-safety trait. Phytoremediation should also be considered separately, because metal accumulation that is advantageous for phytoextraction may be undesirable in food crops.

Metal uptake and accumulation disturb cellular redox and ionic homeostasis, resulting in the production of ROS and changes in Ca^2+^ signaling. These early signals activate MAPK cascades and phytohormonal pathways, which in turn regulate transcriptional responses. The resulting changes in the expression and activity of antioxidant enzymes, chelators, and metal transporters contribute to metal detoxification, intracellular compartmentalization, and maintenance of cellular homeostasis. Thus, chelation, antioxidant defense, MAPK signaling, and hormonal regulation should be considered interconnected components of a coordinated metal-stress response rather than independent defense mechanisms. The relative contribution of these processes depends on metal identity, exposure intensity and duration, tissue type, genotype, and physiological status, and their effects on tolerance and metal accumulation should therefore be interpreted as related but distinct phenotypes.

### 3.1. Metal Chelation and Sequestration

**Figure 1:**
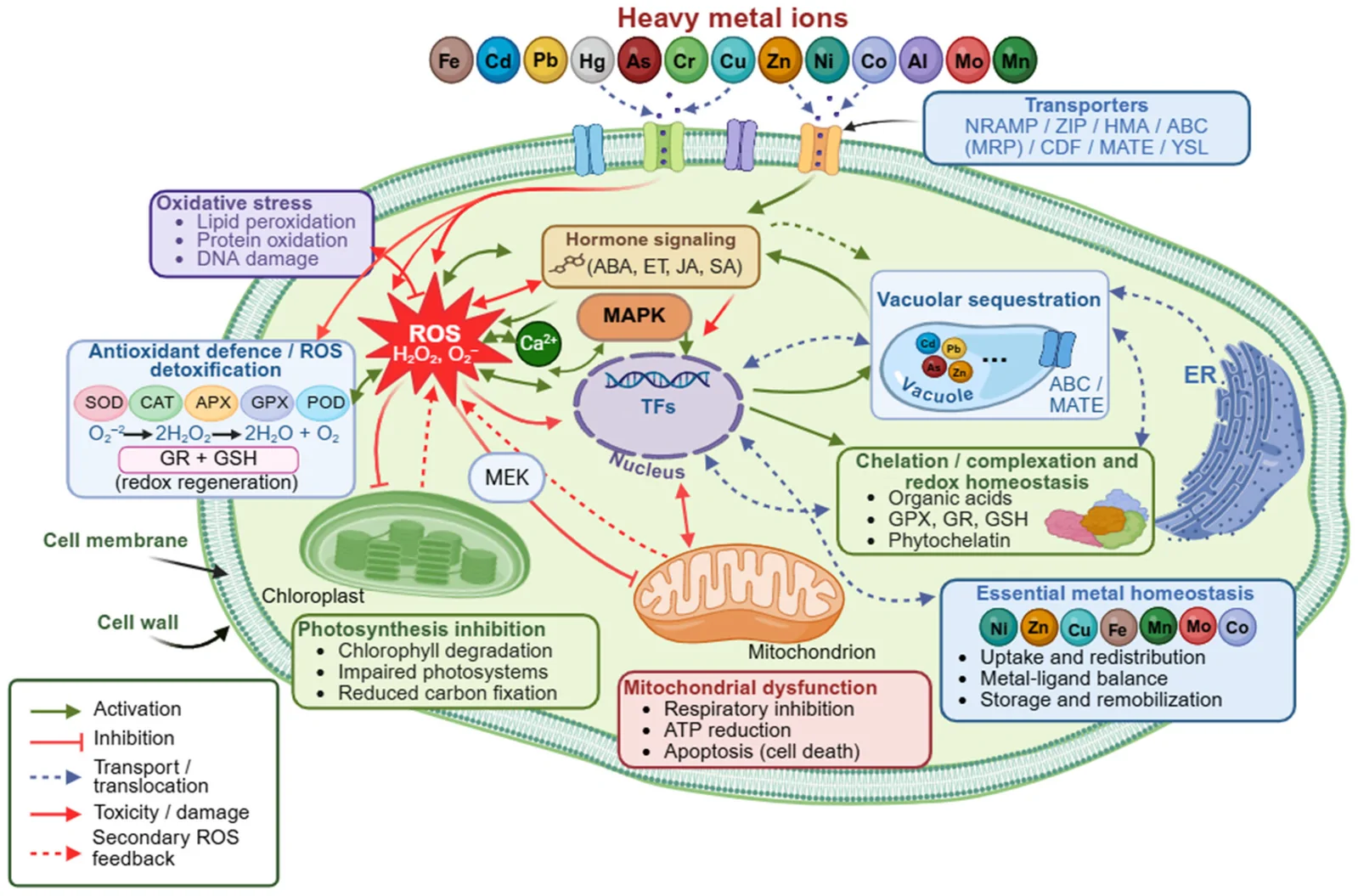
Molecular and physiological mechanisms of plant responses to heavy-metal stress at the cellular level. Heavy metals enter plant cells through membrane transport systems and interact with essential-element transport and homeostasis. Cellular responses include metal chelation by glutathione and phytochelatins, binding to proteins and other ligands, and sequestration in vacuoles. Heavy-metal exposure also induces ROS, leading to oxidative stress and activation of antioxidant defenses. Maintaining redox balance, membrane integrity, ion homeostasis, and cellular metabolism helps protect photosynthetic and metabolic functions and ultimately supports plant growth and tolerance under heavy-metal stress. The black arrows indicate the names of the corresponding cellular structures: the cell wall, cell membrane, and transporter. The figure was created using BioRender.com.

Chelation is one of the primary mechanisms of cellular detoxification of heavy metals in plants. Its function is to bind free metal ions to high-affinity ligands, thereby reducing their cytotoxicity and helping to maintain intracellular ionic homeostasis (Figure 1). The main chelators are phytochelatins (PCs), metallothioneins (MTs), organic acids, and amino acids, which are involved in the binding, detoxification, and intracellular distribution of metals [50].

Metal entry and subsequent accumulation in the cytosol create the conditions for metal binding by these ligands and for activation of downstream detoxification processes. Among the chelation systems, the glutathione (GSH)-phytochelatin pathway is particularly important because GSH serves as a precursor for phytochelatin (PC) synthesis. PC binds metal ions in the cytosol and forms metal–PC complexes, thereby reducing the concentration of free toxic ions. Next, these complexes are transported through the tonoplast into the vacuole, where they are compartmentalized [51,52,53,54,55]. Thus, PC-mediated chelation and ABC-dependent vacuolar sequestration represent two sequential steps of the same detoxification process: metal binding in the cytosol followed by transporter-mediated isolation in the vacuole.

ABC transporters (ATP-binding cassette), also known as MRP (multidrug resistance-associated proteins) for the ABCC subfamily, are among the largest families of membrane transport proteins. In higher plants, the ABC superfamily comprises eight subfamilies: ABCA, ABCB, ABCC, ABCD, ABCE, ABCF, ABCG, and ABCI, whereas the ABCH subfamily, present in some other eukaryotes, is absent in plants [56]. Among them, transporters of the ABCC subfamily are of particular importance in the detoxification of heavy metals because they contribute to the subsequent vacuolar sequestration of metal-PC complexes.

Moreover, the Cre05.g234400 and Cre03.g191350 proteins belong to the ABCG and ABCA subfamilies, respectively, and were identified in *Chlamydomonas reinhardtii* as candidate components of Mo homeostasis associated with nitrate assimilation. Identification of these transporters provides a basis for elucidating their potential roles in Mo homeostasis and nitrate assimilation under metal stress, with implications for nutrient metabolism and metal-stress adaptation [57].

Accordingly, chelation and sequestration function as interconnected downstream components of the metal-stress response. Following metal entry and stress signaling, GSH and PCs reduce the cytosolic availability of toxic ions, whereas transport systems promote their compartmentalization within the vacuole. An important area for further study is the identification of regulatory mechanisms that coordinate chelator synthesis with the expression of genes involved in metal transport and sequestration. From a crop-improvement perspective, genes controlling phytochelatin biosynthesis, metallothionein activity, and vacuolar sequestration represent potential targets for enhancing cellular detoxification and metal tolerance. However, their application in food crops requires simultaneous evaluation of metal uptake, root-to-shoot translocation, and distribution among roots, shoots, and edible tissues, as enhanced intracellular sequestration may improve physiological tolerance without necessarily reducing metal accumulation in edible organs. Therefore, candidate detoxification mechanisms should be evaluated together with yield, essential-element homeostasis, and food-safety traits.

### 3.2. Enzymatic Antioxidant Defense System

Heavy metals can disrupt electron transport, cellular redox homeostasis, and antioxidant metabolism, increasing ROS production. ROS accumulation is therefore both a consequence of metal-induced cellular disruption and a signaling component that activates downstream adaptive responses. Plants counteract this oxidative pressure through coordinated enzymatic antioxidant systems that included superoxide dismutase (SOD), catalase (CAT), ascorbate peroxidase (APX), peroxidase (POD), glutathione peroxidase (GPX), and glutathione reductase (GR) [58,59]. Rather than acting as independent detoxification enzymes, these components form an interconnected redox-control system in which changes in one enzyme may alter the substrate availability and functional requirements of others.

Across different plant–metal combinations, increased antioxidant activity is a recurrent response, but its direction and magnitude are highly context dependent. SOD, POD, CAT, APX, GPX, and GR are frequently induced under Cd, Pb, As, Cr, Cu, Zn, Ni, Co, Mn, and Mo exposure, indicating a broadly conserved requirement for ROS control [60,61,62,63,64,65,66,67,68,69,70,71,72,73,74,75,76,77,78]. However, the antioxidant response is not uniform either across metals or within the same metal treatment. For example, increases in SOD, POD, and CAT under Cd exposure have been reported in some species, whereas different combinations of APX, GR, SOD, and CAT responses occur between leaves and roots of the same plant under comparable stress conditions [60,61]. Similarly, Pb and As exposure can increase several antioxidant activities while suppressing others, and Hg exposure can produce a concentration-dependent transition from enzyme activation at lower concentrations to inhibition at higher concentrations [62,63,64,65,66,67]. These patterns suggest that antioxidant enzyme activity reflects the balance between adaptive ROS detoxification and progressive impairment of cellular metabolism rather than a simple metal-specific response.

Organ specificity and exposure intensity provide important explanations for apparently contradictory observations. Root tissues may exhibit stronger or qualitatively different responses from leaves because they represent the primary site of metal uptake and often experience greater local metal exposure, whereas leaves may respond more strongly to metal translocation and photosynthetic oxidative stress. Likewise, moderate metal exposure may activate antioxidant capacity, whereas prolonged or excessive exposure can impair enzyme activity through oxidative damage, altered cellular redox status, or disruption of protein and cofactor homeostasis. The contrasting responses reported for Hg and As illustrate this transition from adaptive activation to stress-induced inhibition [63,64,65,66,67]. Thus, a decrease in antioxidant enzyme activity should not automatically indicate weaker tolerance; it may instead reflect insufficient induction, enzyme inhibition, or a shift toward alternative protective mechanisms.

The interaction between hormonal signaling and antioxidant defense is further illustrated by Cr(VI) stress. In *Arabidopsis*, Cr(VI) exposure increased the expression of genes involved in ethylene biosynthesis and signaling and was accompanied by enhanced ROS accumulation and downregulation of enzymatic antioxidant-related genes. Inhibition of ethylene signaling or biosynthesis with silver or cobalt reduced Cr(VI)-induced ROS accumulation and alleviated the downregulation of antioxidant-related genes. These findings provide functional support for the involvement of ethylene signaling in Cr(VI)-induced ROS accumulation and its interaction with the antioxidant system, although the contribution of individual antioxidant enzymes to this response requires further validation [68].

The functional significance of increased antioxidant activity also requires careful interpretation. Elevated SOD, CAT, APX, POD, GPX, or GR activity is frequently associated with reduced ROS accumulation and improved physiological performance under metal stress [60,61,62,63,64,65,66,67,68,69,70,71,72,73,74,75,76,77,78,79]. However, enzyme induction alone does not establish that a particular antioxidant enzyme is causally responsible for metal tolerance. Antioxidant activation can represent a general stress response that accompanies, rather than determines, tolerance. Stronger mechanistic evidence is obtained when changes in enzyme activity are supported by genetic manipulation, biochemical inhibition or complementation, ROS measurements, and corresponding tolerance phenotypes. Conversely, observations based solely on enzyme activity or transcript abundance provide evidence of association but do not by themselves establish causal protection against a specific metal.

Importantly, enzymatic antioxidants also interact with non-enzymatic redox systems. GR contributes to the regeneration of reduced glutathione, while APX and related enzymes are functionally connected with the ascorbate–glutathione cycle. Consequently, changes in antioxidant enzyme activity can influence, and be influenced by, glutathione and ascorbate availability, linking enzymatic ROS detoxification with thiol metabolism and metal chelation. The antioxidant response should therefore be considered as an integrated redox-control layer that both limits oxidative damage and contributes to the signaling processes that regulate downstream Ca^2+^, MAPK, hormonal, and transcriptional responses. For crop improvement, antioxidant capacity may represent a useful component trait for maintaining productivity under metal stress, but increased antioxidant enzyme activity alone should not be considered a sufficient breeding criterion. Selection should combine redox-related traits with growth, yield, metal accumulation, and elemental composition.

### 3.3. Non-Enzymatic Antioxidant Defense System

Non-enzymatic components complement the enzymatic antioxidant system and play a direct role in neutralizing ROS and maintaining cellular redox homeostasis. The main components include ascorbate, glutathione, carotenoids, tocopherols, and phenolic compounds [59].

Ascorbic acid (AsA) participates in the neutralization of ROS and the functioning of the ascorbate-glutathione cycle, and is also involved in photosynthesis, cell division, and the biosynthesis of phytohormones. Increased AsA levels help enhance antioxidant defense and plant resistance to abiotic stresses, including heavy-metal exposure [80]. GSH plays a role in maintaining redox homeostasis and metal detoxification; it also serves as a precursor to PCs and participates in conjugation reactions mediated by glutathione-S-transferases (GSTs) [81]. Thus, GSH represents an important functional link between redox control and metal chelation.

Exogenous GSH application has been associated with improved tolerance to Cd, Pb, and Hg by upregulating GSH biosynthesis and/or downregulating transporter genes, thereby limiting metal uptake and translocation [82,83,84,85]. Exogenous sulfur increases GSH levels, promoting PC synthesis and metal chelation, which facilitates their sequestration in root cell walls [86]. These observations further support the close relationship between redox regulation, GSH/PC-mediated chelation, and metal transport. However, these effects do not necessarily demonstrate that GSH acts as the primary determinant of tolerance, because exogenous GSH can simultaneously alter redox status, transporter expression, and thiol metabolism.

Carotenoids and tocopherols primarily protect the photosynthetic apparatus and membrane structures from oxidative damage, whereas phenolic compounds and flavonoids are capable of neutralizing ROS and binding metal ions, thereby reducing their bioavailability [87,88,89]. Additional protective components include selenium and melatonin, which help maintain the antioxidant system and cellular redox homeostasis [50,59,90,91].

Thus, non-enzymatic antioxidants provide an additional level of control over ROS and help maintain redox homeostasis by interacting with enzymatic defenses and chelation mechanisms. Together, enzymatic and non-enzymatic antioxidants form a redox-control layer that also participates in signaling processes leading to downstream transcriptional and metabolic responses. Importantly, ROS generated during metal exposure are not only targets of antioxidant detoxification but also function as signaling molecules that contribute to the activation of Ca^2+^-, MAPK-, hormonal-, and transcriptional responses.

### 3.4. Ca^2+^ Signaling in Plant Heavy-Metal Stress Responses

Ca^2+^ signaling is an early component of the cellular response to heavy metals. Heavy-metal exposure can disrupt calcium homeostasis, with the direction and intensity of the Ca^2+^ signal depending on metal concentration and the metal’s ability to induce ROS formation [92]. At certain concentrations, metals can stimulate hydroxyl radical formation, which activates Ca^2+^-permeable plasma membrane channels and increases cytosolic Ca^2+^ concentration [93]. Cyclic nucleotide-gated channels (CNGCs), glutamate receptor-like channels (GLRs), and two-pore channels (TPCs) may participate in the generation of such Ca^2+^ fluxes [94]. Ca^2+^-binding proteins and sensory systems then perceive changes in cytosolic Ca^2^ and transmit the signal to downstream regulatory pathways. Thus, Ca^2+^ can link early heavy-metal- and ROS-induced changes to the activation of signaling cascades, including MAPK and hormonal pathways. However, at high metal concentrations, excessive stress can disrupt Ca^2+^ fluxes and overwhelm the cell’s buffering capacity, impairing Ca^2+^ signaling and increasing toxicity. Consequently, Ca^2+^ signaling under heavy-metal stress is concentration-dependent: moderate exposure may activate adaptive mechanisms, whereas excessive metal accumulation can disrupt calcium homeostasis and signaling networks [93]. Nevertheless, direct evidence linking specific Ca^2+^ signatures to tolerance against individual metals remains limited, as many studies infer Ca^2+^ involvement from expression-based observations rather than demonstrating causal effects through targeted genetic manipulation.

### 3.5. The Mitogen-Activated Protein Kinase (MAPK) Cascade

The MAPK cascade is a key signaling pathway that enables plants to adapt to heavy-metal stress. Following metal entry and accumulation, changes in ROS, nitric oxide, Ca^2+^, and phytohormone status provide interconnected signals that activate the MAPKKK–MAPKK–MAPK cascade. Sequential protein phosphorylation then transmits these signals to downstream targets, including transcription factors and other regulatory proteins.

Activated MAPKs phosphorylate transcription factors, including WRKY, MYB, bZIP, MYC, and ERF, thereby regulating the expression of genes associated with antioxidant defense, ion transport, detoxification, and compartmentalization of heavy metals. In this way, MAPK signaling provides a mechanistic connection between early stress perception/signaling and the downstream activation of antioxidant systems, chelation, transport, and sequestration. The MAPK integrates stress signals, coordinating nitrogen metabolism, redox homeostasis, and plant defense mechanisms under heavy-metal stress [95,96].

Data on RAF14 and RAF79 in *Chlamydomonas reinhardtii* support the link between MAPK signaling and cellular nitrogen status and the regulation of metabolic and redox responses [97]. Therefore, MAPK cascades can be viewed as signaling intermediates that connect early metal-induced cellular disturbances with transcriptional and metabolic responses.

Although phosphorylation-dependent regulation of downstream targets is a well-established property of MAPK signaling, the specific contribution of individual MAPK modules to tolerance against particular heavy metals remains less consistently resolved. Many studies infer MAPK involvement from changes in transcript abundance and kinase activity, whereas direct substrate identification and genetic validation are available for only a small subset of regulatory relationships. Therefore, MAPK activation should be distinguished from evidence demonstrating a causal MAPK-dependent mechanism of metal tolerance. From a breeding perspective, functionally validated components of Ca^2+^–MAPK signaling may represent potential targets for improving stress responsiveness. However, because MAPK pathways participate in multiple stress and developmental processes, their manipulation may produce pleiotropic effects and should therefore be evaluated in relation to growth, yield, and other agronomic traits.

### 3.6. Hormonal and Signaling Regulation

Phytohormones play a role in plant adaptation to heavy metals by integrating stress perception with growth regulation, redox homeostasis, ion transport, and cellular defense [98,99]. Rather than functioning as independent metal-specific pathways, ABA, auxin, nitric oxide (NO), brassinosteroids, ethylene, salicylic acid (SA), and cytokinins integract with ROS, Ca^2+^, MAPK, and transcriptional networks. The outcome of this crosstalk depends on metal identity, exposure intensity, tissue, developmental stage, and hormonal status. Importantly, hormonal regulation may affect both physiological tolerance and metal distribution, and these outcomes should not be assumed to be equivalent.

Abscisic acid (ABA) is one of the most consistently implicated hormonal regulators of heavy-metal responses. Changes in ABA levels and expression of components of the PYL/PYR/RCAR–PP2C–SnRK2 pathway have been reported under Cd, Hg, Cu, Zn, Pb, Ni, and As exposure [100,101,102,103,104,105,106]. ABA can regulate stomatal closure, water balance, and stress-responsive gene expression and interacts with ROS, Ca^2+^, and MAPK pathways [107,108,109,110]. These relationships provide a mechanistic basis for linking metal-induced cellular distribution with physiological regulation. However, the effects of ABA on metal accumulation are not necessarily uniform, because altered stomatal conductance and root responses can affect both water relations and metal transport.

Auxin and Nitric Oxide (NO) provide a complementary signaling layer, particularly in the regulation of root development and stress adaptation. Heavy metals such as Cd, As, and Cu can disrupt auxin homeostasis, including changes in pin-formed1 (PIN1) activity [111,112,113,114]. Crosstalk between auxin and NO affects root meristem development, whereas exogenous application affecting these pathways has been associated with improved tolerance to Pb, As, and Cd by enhanced antioxidant defense and reduced metal translocation [98,115,116,117,118,119]. These observations support a functional interaction between hormonal and redox signaling; however, responses to exogenous compounds should not be interpreted automatically as evidence that the endogenous auxin–NO pathway is causally responsible for metal tolerance.

NO may also link nitrogen metabolism and stress signaling. The nitrite reductase pathway, involving NO-forming nitrite reductase (NOFNiR), may contribute to NO formation. In *Chlamydomonas reinhardtii*, nitrate reductase (NR) may provide the electron transfer required to reduce nitrite to NO, with the amidoxime reducing component (ARC) participating; conserved ARC genes in *Arabidopsis* suggest a related mechanism in plants [45,46]. However, the direct contribution of this pathway to NO production during heavy-metal stress remains insufficiently established [120]. Thus, the proposed connection between nitrogen metabolism and metal-induced NO signaling should currently be regarded as a mechanistic hypothesis requiring further validation.

Brassinosteroids and SA primarily illustrate the connection between hormonal signaling and redox regulation. Brassinosteroid treatment has been associated with enhanced antioxidant defense and reduced oxidative damage under Ni, Cd, Cu, Zn, Pb, and Cr exposure [69,78,121,122,123,124,125,126,127,128]. Similarly, SA can reduce Cd and Pb accumulation while enhancing antioxidant capacity, glutathione, and PCs metabolism, and other protective responses [129,130,131,132,133,134,135,136]. Cytokinins have also been reported to reduce ROS accumulation and alleviate metal-induced damage [137]. Although these responses are consistent with hormonal modulation of redox homeostasis, much of the available evidence is based on exogenous hormone application, which can produce pleiotropic effects. Therefore, these studies demonstrate the capacity of hormonal manipulation to modify metal-stress responses but do not necessarily establish the endogenous hormonal pathway as the primary determinant of tolerance.

Ethylene provides a more direct example of hormonal crosstalk with signaling and transport networks. Metal exposure has been associated with increased expression of 1-aminocyclopropane-1-carboxylic acid synthase (ACS) and 1-aminocyclopropane-1-carboxylate oxidase (ACO), whereas experimental evidence indicates that interactions between ethylene signaling, MAPK activity, and WRKY33 can contribute to the regulation of ethylene biosynthesis. In addition, ethylene insensitive 2 (EIN2) has been associated with Pb responses and AtPDR12 regulation [138,139,140,141,142,143,144]. Data obtained under Cr(VI) exposure further illustrate the interaction between ethylene and auxin. In *Arabidopsis*, Cr(VI)-induced inhibition of primary root elongation was associated with enhanced ethylene signaling, auxin accumulation in root tips, and increased *AUX1* expression, whereas inhibiting ethylene perception or biosynthesis attenuated these effects [145]. Genotype-dependent responses further confirm the importance of ethylene signaling. In two *O. indica* rice cultivars with differing Cr(VI) sensitivities, the more sensitive cultivar exhibited greater inhibition of root growth and elevated expression of genes related to ethylene biosynthesis and signaling. Inhibiting ethylene perception or biosynthesis reduced the differences in Cr(VI)-induced root growth inhibition between the genotypes, confirming a functional link between ethylene signaling and genotype-specific Cr(VI) sensitivity [146]. Collectively, these results demonstrate how ethylene signaling interacts with auxin, MAPK-dependent transcriptional regulation, and transporter activity, linking stress perception to root growth and heavy metal responses.

Overall, phytohormonal responses to heavy metals are best viewed as an interconnected regulatory layer rather than as independent hormone-specific pathways. ABA strongly contributes to water and stress signaling; auxin and NO link hormonal regulation to root development and redox responses; brassinosteroids, SA, and cytokinins influence antioxidant and protective processes; and ethylene links hormone signaling, MAPK–TF regulation, and transport. However, the strength of evidence varies substantially among these relationships. Physiological responses to exogenous hormones show that hormonal pathways can modify metal-stress responses, whereas genetic manipulation of hormone receptors, biosynthetic components, or downstream signaling elements is needed to establish causal relationships. This distinction is important when interpreting hormonal changes observed in transcriptomic and physiological studies. From a crop-improvement perspective, functionally validated hormonal components may provide opportunities to enhance stress adaptation or modify metal distribution, but their broad effects on growth, development, and reproduction require careful evaluation. Candidate interventions should therefore be assessed not only for improved tolerance but also for metal accumulation, essential-element homeostasis, yield, and quality, particularly in food crops where changes in metal distribution may directly affect food safety.

Taken together, plant responses to heavy metals form an interconnected defense system rather than a sequence of independent mechanisms. Metal-induced disturbances in ionic and redox homeostasis generate ROS, Ca^2+^, NO signals that interact with MAPK and hormonal pathways and ultimately regulate antioxidant capacity, chelation, transport, and intracellular sequestration. However, the relative contribution of each component depends strongly on metal identity, exposure intensity and duration, tissue type, genotype, and physiological state.

An important distinction is therefore needed between mechanisms repeatedly supported across experimental systems and responses that remain primarily associative. Chelation, antioxidant activity, changes in metal transport, and activation of stress signaling are consistently observed under metal exposure; however, their contribution to tolerance cannot always be inferred from changes in metabolite levels, enzyme activities, transcript abundance, or hormone concentrations alone. Stronger causal evidence requires integration of physiological phenotyping with genetic manipulation and direct biochemical or molecular validation, and, where appropriate, complementary multi-omics analyses.

## 4. Transcription Factors and Metal Transport Systems Involved in Heavy-Metal Stress Responses

**Figure 2:**
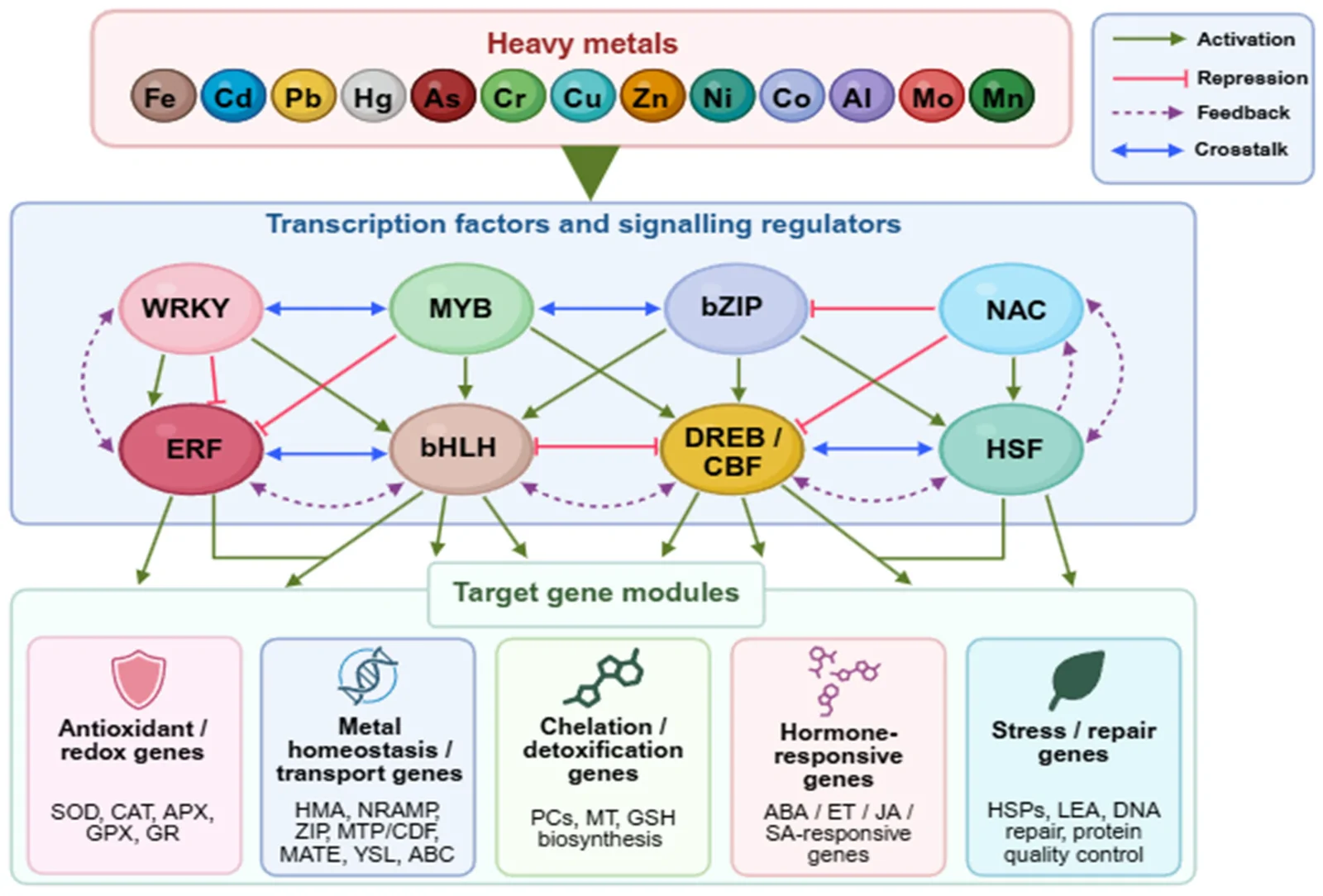
Regulatory and evidence-based networks underlying plant responses to heavy-metal stress. The figure illustrates interconnected regulatory networks involving major transcription factor families, including WRKY, MYB, bZIP, NAC, ERF, bHLH, DREB/CBF, and HSF, which regulate metal transport and sequestration, antioxidant and detoxification responses, redox homeostasis, and stress adaptation. Solid and dashed connections distinguish experimentally supported relationships from proposed or associative interactions. The figure was created using BioRender.com.

Transcription factors (TFs) do not act independently in plant responses to heavy metals but function as interconnected regulatory networks that integrate redox, ionic, mitogen-activated protein kinase (MAPK), and hormone-dependent signals with antioxidant defense, metal transport, chelation, and intracellular sequestration (Figure 2). Heavy-metal exposure can induce ROS accumulation, disrupt ion homeostasis, and activate signaling cascades that modulate TFs belonging to the WRKY, MYB, bZIP, NAC, ERF, bHLH, and DREB/CBF families [147,148,149,150,151,152,153,154]. These TFs regulate overlapping sets of downstream genes involved in redox homeostasis, metal uptake and transport, glutathione and phytochelatin metabolism, and vacuolar compartmentalization. However, their effects are not uniform across species, metals, tissues, or experimental conditions. Thus, TF-mediated heavy-metal responses are better interpreted as interconnected regulatory modules rather than as independent family-specific functions.

### 4.1. Stress-Signaling Networks Connecting ROS and MAPK Pathways with Transcriptional Regulation

Heavy-metal exposure rapidly alters cellular redox and ionic status, leading to ROS accumulation and activation of stress-signaling cascades. ROS can function not only as damaging molecules but also as signaling components that activate MAPK pathways and hormone-responsive transcriptional networks. WRKY, NAC, ERF, bZIP, and DREB/CBF proteins can act downstream of or interact with these signaling pathways to regulate stress-responsive genes [147,149,150,151,152,153,154]. The resulting transcriptional response integrates antioxidant protection, ion homeostasis, metal transport, and cellular repair.

The WRKY family provides an important example of this signaling architecture. WRKY proteins can be activated through MAPK-dependent pathways and bind W-box elements in the promoters of stress-responsive genes, thereby linking upstream stress signaling with downstream antioxidant and metal-homeostasis responses [147]. Experimental studies show that individual WRKY proteins can either enhance or attenuate metal tolerance, depending on the biological context. For example, TaWRKY74, ZmWRKY4, AtWRKY45, and StWRKY6 have been associated with enhanced tolerance to heavy metals, whereas AtWRKY12, AtWRKY46, ScWRKY35, and CaWRKY41 have been reported to negatively regulate components of the defense response [155]. These contrasting effects indicate that WRKY-mediated regulation is context-dependent and cannot be generalized across the family. The outcome may depend on plant species, tissue, developmental stage, metal type, and the TF’s position within the signaling network.

NAC and ERF proteins provide additional links between stress signaling and transcriptional regulation. NAC proteins interact with ABA- and MAPK-dependent pathways and regulate genes involved in ROS detoxification, ion homeostasis, and metal-responsive defense [149,156,157,158,159]. Similarly, ERF proteins represent important transcriptional outputs of ethylene signaling and regulate genes associated with antioxidant defense, ion transport, and stress adaptation [150,160]. Thus, WRKY, NAC, and ERF proteins should be viewed as interconnected transcriptional nodes that integrate ROS, MAPK, and hormone signals rather than as independent regulators of heavy-metal tolerance.

### 4.2. Transcriptional Networks Regulating Antioxidant and Redox Homeostasis

A major transcriptional response to heavy-metal stress involves maintaining redox homeostasis. Excessive metal accumulation can stimulate ROS production, whereas ROS-responsive TF networks regulate antioxidant enzymes and thiol metabolism to limit oxidative damage. WRKY, MYB, bZIP, NAC, and ERF proteins have all been associated with regulating genes involved in antioxidant defense and glutathione metabolism [147,148,149,158,159,160,161,162].

Experimental evidence supports a role for specific TFs in this network. For example, overexpression of *SpbZIP60* increased Cd tolerance in transgenic *Arabidopsis* plants by reducing ROS accumulation, protecting photosynthetic performance, and decreasing Cd content [163]. Similarly, overexpression of *TmMYB16/123*, *DcMYB62*, *PsMYB62*, and *SbMYB15* has been associated with enhanced tolerance to Cd and Ni, accompanied by increased SOD, POD, and CAT activities, reduced ROS accumulation, and maintenance of photosynthetic activity [161,162]. These findings indicate that manipulation of individual TFs can modify the capacity of plants to maintain redox balance under metal stress.

However, these observations should not be interpreted as evidence that individual TFs universally activate antioxidant defenses. The transcriptional effects of a given TF may vary according to the metal, concentration, plant species, tissue, developmental stage, and genetic background. Moreover, in some studies, the proposed regulatory relationship is inferred from differential expression or overexpression phenotypes rather than demonstrated through direct TF–promoter binding. Therefore, experimentally validated direct regulatory interactions should be distinguished from associations identified through transcriptomic or physiological analyses.

### 4.3. TF-Mediated Regulation of Metal Uptake, Transport, and Homeostasis

Another major regulatory module links TF activity with the expression of metal transport and homeostasis genes. The balance between metal uptake, intracellular distribution, and sequestration determines the extent of metal accumulation in sensitive cellular compartments. MYB and bHLH proteins are particularly relevant to this network because they regulate components of mineral-nutrient signaling and transporter expression.

An experimentally supported example is provided by the *Arabidopsis* MYB–bHLH regulatory module. AtMYB49 promotes Cd uptake and accumulation by regulating *bHLH38/101* and downstream genes, including *IRT1*, *HIPP22*, and *HIPP44*. This finding shows how TF interactions can link transcriptional regulation to transporter activity and ultimately determine Cd accumulation. Conversely, other MYB proteins, including AtMYB4 and AtMYB75, have been associated with Cd detoxification and vacuolar compartmentalization, indicating that members of the same TF family can have opposing effects on metal homeostasis [148].

bHLH proteins also participate in the regulation of Fe homeostasis, which is closely connected with Cd responses. Because Cd can interfere with Fe acquisition and utilization, transcriptional regulation of Fe-deficiency responses may indirectly affect Cd toxicity. Members of the bHLH family regulate genes associated with metal transport, nicotinamide synthesis, Fe homeostasis, and antioxidant defense, and contribute to tolerance to Al, Mn, and other metals [148,164]. Qian et al. identified 32 bHLH subfamilies and reported the involvement of subfamily 4, including ZmbHLH105, in responses to heavy-metal exposure. Nevertheless, the specific contribution of individual bHLH subfamilies to the transport and tolerance of particular metals remains insufficiently resolved [164].

These observations emphasize that TF-mediated regulation of metal transport is embedded within broader nutrient-homeostasis networks. Because many metal transporters have broad substrate specificity, changes in their expression may affect several metals simultaneously, making it difficult to assign a specific TF–transporter interaction exclusively to resistance against a single metal.

### 4.4. Transcriptional Regulation of Metal Chelation and Intracellular Sequestration

TF networks also connect metal sensing and signaling with intracellular detoxification mechanisms. Following metal uptake, plants can reduce the cytosolic availability of toxic ions by binding them to glutathione-derived ligands, phytochelatins (PCs), metallothioneins (MTs), and other chelators, followed by transport and sequestration in vacuoles or other cellular compartments. WRKY, MYB, bZIP, and NAC proteins have been associated with the transcriptional regulation of genes involved in glutathione metabolism, PC and MT synthesis, and metal sequestration [147,148,158,159].

In this network, TF-mediated changes in glutathione metabolism may influence the availability of substrates required for phytochelatin synthesis and consequently affect metal binding. Similarly, regulation of transporter genes involved in vacuolar sequestration can determine the intracellular distribution of metal–ligand complexes. These processes provide a functional connection between transcriptional regulation, thiol metabolism, and cellular compartmentalization. Nevertheless, evidence for direct transcriptional control of specific chelation or sequestration genes varies considerably among TFs.

### 4.5. Hormone–TF Crosstalk in Heavy-Metal Responses

Hormonal signaling provides an additional layer of integration within transcriptional networks responding to heavy metals. ABA, ethylene, salicylic acid, and other phytohormones interact with ROS- and MAPK-dependent pathways and influence the activity of TFs involved in stress adaptation. NAC proteins are closely connected with ABA and MAPK signaling, whereas ERF proteins function predominantly within ethylene-responsive regulatory pathways [149,150,160,163].

ERF-dependent responses are particularly evident under Cd exposure. Members of the ERF family show differential expression in Arabidopsis, rice, wheat, eggplant, and poplar following Cd treatment, and several studies have associated ERF activity with antioxidant defense, stress signaling, and cellular homeostasis [148,165,166,167,168,169,170]. These observations support the involvement of ERF-mediated transcriptional regulation in Cd responses. However, differential expression alone does not establish that a particular ERF directly controls metal tolerance, and the contribution of individual ERF members may depend on their interaction with ethylene, ROS, MAPK, and other signaling pathways.

Similarly, NAC proteins respond to Cd, Pb, As, Cu, and other toxic metals and regulate genes associated with ROS detoxification, antioxidant defense, ion homeostasis, and metal-binding processes [149,156,157,158,159]. Their interactions with ABA and MAPK signaling suggest that NAC-dependent responses may represent a broader abiotic-stress network rather than a metal-specific pathway. Functional redundancy among NAC proteins and their dependence on species, tissue, developmental stage, and metal type further complicate the assignment of specific functions to individual members.

### 4.6. DREB/CBF and HSF Networks as General Stress Response Modules

DREB/CBF and heat shock factors (HSFs) represent additional transcriptional modules that may contribute to heavy-metal responses, although their functions appear to be less metal-specific than those of some transport- or detoxification-associated regulatory pathways. DREB/CBF proteins regulate broad abiotic-stress responses through binding to DRE/CRT elements in the promoters of stress-responsive genes [151,152,153,154]. In *Ricinus communis*, Cd exposure induced *DREB-1B*, *DREB-1F*, and *CBF* expression, while their overexpression increased tolerance to the toxic effects of heavy metals [171]. These findings support a functional contribution of DREB/CBF signaling, but do not necessarily indicate a metal-specific regulatory mechanism.

Additional evidence comes from *Cannabis sativa*, where foliar application of Mo under Cd stress enhanced CBF/DREB expression together with increased proline and polyphenol accumulation [172]. Positive associations among CBF/DREB expression, Cd accumulation, proline, and polyphenol content suggest that this transcriptional module may participate in the physiological response to Cd. However, these correlations do not establish a direct causal role of CBF/DREB in Mo-mediated Cd tolerance. Their contribution may instead reflect activation of a broader abiotic-stress response that subsequently influences osmotic adjustment, antioxidant protection, and cellular damage limitation [172,173,174]. Therefore, DREB/CBF should be considered part of a general stress-signaling network unless experimental evidence demonstrates direct metal-specific regulatory interactions.

HSFs provide another example of a general stress-response pathway. HSF1 activation under heavy-metal stress can induce heat shock proteins such as HSP70/HSP72, which contribute to protein homeostasis and cellular protection by stabilizing or repairing stress-damaged proteins [175]. However, current evidence primarily supports HSPs as components of the cellular stress-response machinery rather than as direct mediators of metal detoxification. Thus, HSF-dependent regulation may contribute indirectly to heavy-metal tolerance by maintaining proteostasis during prolonged stress.

Overall, current evidence indicates that TF-mediated responses to heavy metals are organized into interconnected regulatory networks rather than isolated family-specific pathways. ROS and Ca^2+^ signaling, MAPK cascades, and hormone-dependent pathways converge on TFs that converge on several major processes, including antioxidant defense, metal transport, glutathione and phytochelatin metabolism, and intracellular sequestration. Across different plant systems, the magnitude and direction of individual TF responses vary with metal identity, concentration, exposure duration, tissue, genotype, and environmental conditions, indicating substantial context dependence rather than uniformly conserved responses. Importantly, evidence strength differs among these regulatory relationships: differential expression, correlation, and co-expression provide evidence of association but do not establish causality, whereas direct TF–promoter interactions and genetic loss- or gain-of-function studies combined with target validation provide stronger mechanistic support. Overexpression-associated phenotypes should also be interpreted cautiously because ectopic expression may produce pleiotropic effects. Future studies combining ChIP-seq, DAP-seq, promoter-reporter assays, CRISPR-based functional validation, and multi-omics approaches will be essential for resolving the hierarchy, target specificity, and context dependence of these transcriptional networks under different metal stresses. From a crop-improvement perspective, functionally validated TFs represent potential targets for breeding or genome editing because they can coordinate multiple downstream stress-response processes. However, their broad regulatory activity may also produce pleiotropic effects on growth, development, and yield. Therefore, candidate TFs should be evaluated not only for enhanced stress tolerance but also for metal accumulation, essential-element homeostasis, agronomic performance, and stability across environments, particularly in food crops where improved physiological tolerance should not come at the expense of food safety.

## 5. Transcriptomic Studies in Plants

Transcriptomic analyses have substantially expanded our understanding of the molecular mechanisms underlying plant responses to heavy-metal stress. Across different species and experimental conditions, RNA-seq studies have identified recurrent transcriptional modules associated with metal transport and sequestration, redox homeostasis, signaling, transcriptional regulation, and metabolic and cell-wall remodeling (Table 1 and Table S2).

### 5.1. Scale and Temporal Dynamics

One of the most consistent observations across transcriptomic studies is the coordinated activation of several functional modules rather than isolated responses in individual genes. A temporal reorganization of these modules has been particularly well documented under Cd and Cr stress. In *Vigna radiata*, transcriptomic profiles changed substantially after 1, 5, and 9 days of Cd exposure, whereas in *Paspalum vaginatum,* the transcriptional response expanded with exposure duration from 1 to 24 h. The responsive genes were predominantly associated with metal transport, glutathione metabolism, and transcriptional regulation [176,177]. Similarly, Cd exposure in *Populus × canadensis* affected genes associated with oxidoreductase activity, secondary metabolism, and stress response, while Cd stress in the roots of *Arundo donax* activated genes involved in antioxidant defense and ethylene signaling [178,179].

Cr stress further illustrates the temporal coordination of distinct molecular processes. In *Canna indica*, transcriptomic changes were characterized by predominant alterations in carbohydrate metabolism at the early stage, followed by changes in hormonal and MAPK signaling at the intermediate stage, and activation of glutathione metabolism and phenylpropanoid biosynthesis at the late stage [180]. Thus, transcriptomic data support a dynamic response in which early metabolic and signaling changes are followed by enhanced detoxification and protective metabolism.

Responses to multiple metals also reveal recurrent transcriptional components. In *Camellia sinensis*, exposure to Cd and As resulted in changes in genes associated with stress responses, including an ERF transcription factor and structural genes potentially involved in resistance to both metals [181]. Meanwhile, in *Medicago truncatula* Cd- and Hg-responsive expression patterns were tissue-, genotype-, and metal-specific, demonstrating that the conserved components of the response are modified according to the biological and chemical context [182].

### 5.2. Metal Transport, Chelation, and Sequestration

One of the most consistently observed features of the transcriptomic response to heavy-metal stress is the regulation of metal uptake, transport, and intracellular redistribution, and sequestration. Transcriptomic studies consistently implicate transport and sequestration mechanisms.

In *Phytolacca americana*, Cd-responsive transcripts included genes encoding MTs, aquaporins, and ABC transporters [183]. In *Gossypium hirsutum*, ABC, CDF, and HMA transporter genes were differentially expressed, and functional analysis indicated that GhHMAD5 contributed to Cd tolerance, providing evidence beyond transcriptomic association alone [184]. Similarly, Cd exposure in *Cannabis sativa* induced HMA genes involved in Cd transport and sequestration, while Cd-responsive transcripts in *Siegesbeckia orientalis* included NRAMP5, HMA, ABC transporter 1, and pleiotropic resistance proteins, as well as genes associated with glutathione and antioxidant metabolism [185,186].

Transcriptomic analysis also identified transport-related responses in microalgae. In *Chlamydomonas reinhardtii*, a genome-wide, evolutionary, and transcriptomic analysis identified 75 ABC proteins belonging to eight subfamilies. Their transcriptional and promoter profiles suggested potential roles in heavy-metal detoxification, lipid metabolism, and environmental adaptation, highlighting ABC transporters as a conserved component of stress-responsive metal homeostasis in microalgae [187].

Several studies further link transporter expression with differences in metal accumulation and sequestration. In *Zea mays*, genes associated with low Cd accumulation were mainly involved in SAM metabolism, metal transport, and vacuolar sequestration [188]. In *Oryza sativa*, approximately Cd-responsive transcripts included metal transporter genes whose expression dependent on Cd concentration [189]. In *Hordeum vulgare*, the HvPCR2 genes, involved in Cd detoxification, were identified, whereas YSL12 was implicated in heavy metal transport in *Triticum aestivum* [190,191]. In *Phoenix dactylifera*, transporter and chelator genes became particularly responsive after prolonged Cd exposure, consistent with enhanced metal binding, transport, and detoxification [192]. In *Solanum nigrum*, differences in the expression of metal-transporters and Fe homeostasis-related transporters were associated with variation in Cd uptake and redistribution [193].

Responses to Al further demonstrate the importance of transporter networks. In *Hydrangea macrophylla*, Al stress induced root genes, including MATE, ABC, and NRAMP transporters [194]. In *Fagopyrum* species, Al tolerance was associated with ART1/STOP-dependent regulation and transport systems involving STAR1/STAR2, ALS1/ALS3, MATE, and FtFRDL [195,196]. These responses indicate that Al tolerance involves coordinated regulation of transport and detoxification mechanisms rather than a single transporter pathway.

Pb stress also activates overlapping transport and detoxification pathways. In *Medicago sativa*, Pb-responsive DEGs included ABC, IRT, and CDF transporters, whereas in *F. tataricum* MTPC2, PCSL, VCE1a, and NRAMP3 were differentially expressed [197,198]. In *Ligusticum chuanxiong* and *Triticum aestivum*, ATPases, MSR2, HAM3, and ABC transporter genes were associated with transport, sequestration, and detoxification [199,200]. Moreover, 33 BjMTP genes in *B. juncea* displayed subgroup-specific responses to Mn, Fe, Zn, Cd, Sb, and Pb, demonstrating that members of the same transporter family can respond differentially to individual metals [201].

The close relationship between metal toxicity and nutritional homeostasis is also evident at the transcriptomic level. Under Zn stress in *P. canadensis*, ZIP2 and NRAMP1 genes were associated with Fe transport and cellular homeostasis, together with pathways involved in metal chelation and detoxification [202]. In *C. sinensis* exposed to Al, Cu, and Cd, numerous transcriptional changes included metal transporter genes whose expression varied among cultivars and was associated with differences in metal accumulation [203]. Comparative analysis of *Arabidopsis thaliana* and *A. halleri* under Cd and Cu stress also revealed common, metallophyte-specific, and Cd-specific transcriptional responses, including candidate genes associated with Cd hypertolerance and signaling changes related to Zn deficiency [204].

Non-coding RNAs may provide an additional regulatory layer linking transcriptional regulation with metal transport and detoxification. In *H. vulgare* under Cd stress, 26 lncRNAs and 150 mRNAs associated with Cd tolerance were identified, including transcripts potentially involved in ABC transporter regulation, stress response, and detoxification. Twelve key lncRNAs formed 18 predicted lncRNA–mRNA regulatory pairs, suggesting that non-coding RNA-mediated regulation may contribute to the coordination of metal transport and detoxification [205].

Collectively, these studies identify metal transport, chelation, and sequestration as a conserved transcriptional module, while also demonstrating substantial specialization among transporter families and individual genes. Importantly, differential expression alone does not establish a causal role in metal tolerance. Functional genetic and physiological studies are therefore required to distinguish genes that merely respond to metal exposure from those that directly contribute to metal exclusion, redistribution, detoxification, or sequestration.

### 5.3. Antioxidant and Redox Responses

Redox regulation represents another conserved molecular module of the transcriptomic response to heavy-metal stress. Transcriptomic studies complement biochemical observations by demonstrating coordinated changes in genes associated with antioxidant enzymes, glutathione metabolism, metal transport, and redox signaling.

In *Solanum aculeatissimum* under Cd stress, the expression of genes encoding HMA-ATPase, phytochelatin, POD, and ascorbate oxidase genes was altered. Increased antioxidant enzyme activity and expression of SaHMA20, SaL-AO, SaPrxs4, and SaPCs were associated with Cd detoxification and hyperaccumulation [206]. In *Gossypium* spp., Cd exposure was accompanied by enhanced antioxidant defense together with changes in metal transport and flavonoid biosynthesis [184,207]. In *H. vulgare*, Cd stress altered the expression of CAT genes and other stress-associated components, including ABC transporters, cytochrome P450, and V-ATP synthase [208].

Transcriptomic and physiological responses can also vary according to exposure duration. In *Z. mays* exposure to Pb, SOD activity increased during the first 12–48 h, whereas POD activity showed the opposite trend [209]. Similarly, in *Arachis hypogaea* L., exogenous SCC increased SOD activity, while reducing malondialdehyde (MDA) levels, indicating modulation of oxidative stress responses [210]. In *Pogonatherum crinitum* under Pb stress, resistance genes associated with antioxidant defense, metal transport, and transcriptional regulation were identified; their increased expression correlated with the activation of SOD, CAT, and AsA [211].

The integration of redox responses with signaling pathways is also evident in Chinese cabbage, where Cd and Pb exposure altered the expression of genes encoding antioxidant enzymes, MAPK signaling components, and calmodulin, indicating coordinated activation of oxidative stress and signaling responses [212]. In response to As exposure in *A. thaliana*, genes associated with heat and oxidative stress—including HSPs, HSFs, ubiquitin, and aquaporins—as well as components of the ethylene signaling pathway were identified [213]. A candidate LRR-RLK VIII gene associated with As resistance and root accumulation was also identified. In *O. sativa*, exposure to Cd and As altered genes associated with redox control, stress response, transcriptional regulation, transmembrane transport, signal transduction, and sulfur compound metabolism [214].

Metal-specific redox modules have also been observed under Cu and combined-metal stress. A hyperaccumulating ecotype of *B. nigra* exhibited strong induction of genes encoding the glutathione pathway, metal-ATPases, and ABC transporters under Cu stress [215]. Under combined Co+Cu stress, the MAPK signaling and glutathione pathways were activated in a tolerant genotype of *H. vulgare* [216].

Together, these findings indicate that transcriptional regulation of antioxidant and glutathione metabolism is closely integrated with metal transport, signaling, and detoxification. Thus, the redox response should not be considered an independent downstream reaction but rather as a component of a broader transcriptional network coordinating ROS homeostasis with metal handling.

### 5.4. Hormonal, Ca^2+^, and MAPK Signaling

Transcriptomic studies further demonstrate that hormonal and MAPK signaling coordinate growth, stress response, and metabolic reprogramming. In *Brassica* spp., transcriptomic analysis identified genes associated with ion transport and auxin signaling [217]. In A. *hypogaea*, exogenous SCC under Cd stress increased the expression of AUX/IAA, GH3, SAUR, and JAZ genes associated with root growth regulation and the hormonal defense response [210]. In *Hibiscus cannabinus*, Cd exposure activated antioxidant enzymes, MAPK signaling, and detoxification-related genes [218]. In *Salix babylonica* exposed to Co, tissue-specific transcriptional responses were associated with antioxidant defense, phytohormone signaling, Co detoxification, and the maintenance of ion homeostasis [219].

Hormonal responses are also metal-specific. Under Al stress in *Z. mays*, genes associated with auxin, gibberellic and jasmonic acids, and brassinosteroids were differentially expressed [220]. Cd stress was accompanied by the activation of hormonal signaling together with changes in genes related to antioxidant defense and the cell wall [200]. Under Cr stress in *C. indica*, changes in hormonal and MAPK signaling predominated at the intermediate stage, whereas in *O. sativa* under Cr(VI) stress, the ethylene, cytokinin, and MAPK pathways, as well as CDK and the ubiquitin-proteasome system, were altered [180].

Ca^2+^-associated signaling also emerges as an important component of the transcriptomic response. In *S. alfredii* exposed to Cu, transcriptomic analysis identified the Ca^2+^-signaling proteins CML19 and CDPK34/17, together with changes in the expression of HMA5, NRAMP3, VIT1, and ALMT9, suggesting coordinated regulation of Ca^2+^ signaling and metal homeostasis [221]. These observations are consistent with the integration of Ca^2+^-dependent signaling with transcriptional regulation and metal transport.

Overall, transcriptomic evidence indicates that hormonal, Ca^2+^, and MAPK pathways are not activated independently but are integrated with redox regulation, transport, and transcription-factor networks. Their relative contribution depends on the metal, exposure conditions, tissue, and genotype.

### 5.5. Primary and Secondary Metabolism

Heavy-metal stress also induces extensive transcriptional reprogramming of primary and secondary metabolism, providing metabolic support for defense processes and contributing to the synthesis of compounds involved in metal binding and detoxification.

In *H. vulgare* under Cd stress, the expression of carbohydrate metabolism genes was altered, as well as that of genes associated with defense proteins, dehydrin, ABC transporters, cytochrome P450, V-ATP synthase, expansin, and α-xylosidase [208]. In *Pinus banksiana* under Ni stress, transcriptional changes were predominantly associated with the stress response, carbohydrate metabolism, and catabolic processes [222]. In *O. sativa*, exposure to Cd, As, Pb, and Cr was accompanied by changes in secondary metabolism, flavonoid biosynthesis, lipid, amino acid, and carbohydrate metabolism, ascorbate and alderate metabolism, xenobiotic biodegradation, membrane transport, ABC transporters, GST, and MAPK signaling [223].

Changes in metabolism are also closely associated with transporter and antioxidant responses. Under Al stress in *F. tataricum*, changes in antioxidant activity and lipid metabolism were observed alongside transporter regulation, whereas in *Z. mays,* Al stress, affected genes associated with the tricarboxylic acid cycle [195,220]. In *Festuca arundinacea*, exposure to graphene oxide was accompanied by an upregulation of energy, terpenoid, polyketide, and carbohydrate metabolism pathways, as well as zeatin biosynthesis and the degradation of limonene and pinene [224].

Secondary metabolism pathways, particularly phenylpropanoid and flavonoid biosynthesis, may provide compounds with antioxidant and chelating properties. In *Cunninghamia lanceolata*, Al exposure altered secondary metabolism, including flavonoids and phenylpropanoids biosynthesis [225]. In *Verbena bonariensis*, Cd stress was associated with changes in genes involved in lignin, CHS, and ANS synthesis, indicating the activation of phenylpropanoid and flavonoid metabolism [226]. A similar activation of secondary and phenylpropanoid metabolism was observed in Chinese cabbage upon exposure to Cd and Pb [212]. Under combined Co+Cu stress, the phenylalanine, anthocyanin, and flavonoid metabolic pathways were activated in a resistant genotype of *H. vulgare* [216].

Thus, transcriptomic evidence indicates that metabolic reprogramming constitutes an integral component of heavy-metal adaptation and is functionally connected with redox regulation, metal binding, transport, and cellular remodeling.

### 5.6. Tissue-, Genotype-, and Metal-Specific Responses

Although several molecular modules are recurrent across transcriptomic studies, their activation is strongly dependent on tissue, genotype, metal identity, and exposure duration. This specificity is particularly evident in genes associated with the cell wall, which can contribute to limiting the movement of toxic metals within plant tissues. In *A. donax*, Cd stress activated genes involved in lignification and cell wall reinforcement, while similar changes in cell wall-related transcripts were observed in wheat and *V. bonariensis* under Cd exposure [179,200,226]. In *C. lanceolata*, Al stress significantly altered the metabolism of cell wall components, whereas in *Glycine max*, genes associated with cellulose production, including cellulose synthases, were identified as potentially involved in resistance to Al [225,227].

### 5.7. Transcription Factors and Regulatory Networks

Transcription factors provide an important regulatory layer linking signaling pathways to the expression of genes involved in metal transport, antioxidant defense, signaling processes, and the reorganization of cellular metabolism. Transcriptomic studies have identified multiple TF families, including WRKY, bHLH, ERF, bZIP, MYB, HSF, C2H2, AP2/ERF, and GARP, in plants exposed to different heavy metals [197,209,221,225,228,229,230].

In resistant *G. max* varieties, Cd stress was associated with activation of ABC transporters, together with WRKY and bHLH factors [228,229]. In *M. sativa* under Pb stress, WRKY, ERF, and bZIP factors were identified together with genes encoding POD, SOD, GST, flavonoid and isoflavonoid metabolic enzymes, and ABC, IRT, and CDF transporters [197]. In *C. lanceolata* under Al stress, bHLH, C2H2, ERF, bZIP, GRAS, and MYB transcription factors were associated with stress adaptation, secondary metabolism, and cellular remodeling [225]. In *C. sinensis*, an ERF transcription factor was identified under Cd and As stress, potentially involved in general resistance to both metals [181]. In *S. alfredii*, transcriptomic analysis identified 649 transcription factors, predominantly belonging to zinc finger, MYB, bHLH, and bZIP families, indicating extensive transcriptional regulation of the stress response [230].

**Table 1:** Transcriptomic responses, molecular pathways, candidate genes, and functional evidence associated with heavy-metal stress in plants.

Plant Species	Metal	Dose	Exposure Duration, Tissue	Key Pathway	Candidate Genes	Functional Validation
*P. vaginatum*	Cd	300 μmol/L	0, 1, 4 and 24 h; leaves	Metal transport; glutathione metabolism; transcriptional regulation	*PvSnRK2.7*	Functionally supported [177]
*C. sinensis*	Cd, As	120 mg/kg	10, 15 days; roots	Stress signaling; transcriptional regulation	ERF; *CSS0004428*	Candidate association; validation not reported [181]
*G. hirsutum*	Cd	0.5, 1, 2, 4, 6, 8 and 10 mM	1, 3, 6, 9, 12, 24 h; leaf, stem and root	Metal transport; redox response; tolerance mechanism	*GhHMAD5*	Functionally supported: GhHMAD5 contributed to Cd tolerance [184]
*H. vulgare*	Cd	80 μM	5 days; shoot and root	Detoxification	*HvPCR2*	Candidate gene; functional validation not reported [190]
*T. aestivum*	Cd	0, 1, 3, and 10 mg/L	0, 2, 6, 12, 24, 48, and 72 h; root	Amino acid metabolism, ABC transporter activity, plant hormone signal transduction, phenylalanine metabolism, and flavonoid biosynthesis	*YSL12*; *TraesCS2B02G394500TraesCS2B02G394500*	Candidate gene; functional validation not reported [191]
*F. esculentum*	Al	0 or 20 μM	3 days; shoot and root	STOP/ART1-dependent Al tolerance; organic-acid transport	STOP1/ART1, FeSTAR1, FeALS3, FeALS1, FeMATE1 and FeMATE2	Candidate association; validation not reported [196]
*F. tataricum*	Al	0.5 mM	24 h; root	ART1/STOP signaling; citrate transport; organic acid metabolism; antioxidant and detoxification pathways	STAR1, STAR2/ALS3, ALS1, FtFRDL1, FtFRDL2	Candidate association; validation not reported [195]
*L. chuanxiong*	Cd	0, 5, 10, 20, 40 mg/L^−1^		Hormonal signaling; antioxidant defense; cell-wall modification; transport/sequestration	MT genes	Transcriptomic/physiological association; validation not reported [199]
*A. thaliana*	Cd, Cu	10, 50 μM Cd), 10 μM (Cu)	10–12 days; root	Metal-specific signaling; Zn deficiency; Cd hypertolerance	Candidate Cd-tolerance genes	Candidate association; validation not reported [204]
*A. halleri*	Cd, Cu	25, 125 μM Cd), 10 μM (Cu)	10–12 days; root	Metal-specific signaling; Zn deficiency; Cd hypertolerance	Candidate Cd-tolerance genes	Candidate association; validation not reported [204]
*H. vulgare*	Cd	20 μmol L^−1^	24 h; root	lncRNA–mRNA regulation; ABC transport; detoxification	26 lncRNAs; 150 mRNAs	Regulatory association; functional validation not reported [205]
*S. aculeatissimum*	Cd	3, 10, 20, 30, 40, 50 40 mg/kg^−1^	90, 163 days; stem, leaves, and root	HMA-mediated transport; phytochelatin; antioxidant defense	*SaHMA20*, *SaL-AO*, *SaPrxs4*, *SaPCs*	Functionally associated with Cd detoxification/hyperaccumulation [206]
*A. hypogaea*	Cd	2.5 mg/kg	15 days, leaves	Antioxidant defense; cell wall biosynthesis; secondary metabolism; plant hormone signal transduction	AUX/IAA, GH3, SAUR, JAZ	Physiological/transcriptomic evidence; causal validation not reported [210]
*A. thaliana*	As	100 to 300 μM	2 days; root	Oxidative/heat stress; ethylene signaling	LRR-RLK VIII; HSPs; HSFs	Candidate LRR-RLK VIII associated with As tolerance; functional validation not reported [213]
*G. max*	Al	25 μM	6, 12 or 24 h; root	Cellular glucan metabolic process; regulation of transcription	*Glyma.02G205800*	Functional validation not reported [227]
*G. max*	Cd	75 μmol·L^−1^	7 days; root	Antioxidants; sequestration; metal-transporting	*GmHMA5*	Functional validation [228]
*S. alfredii*	Cd, Zn, Pb, Cu	100 μM (Cd, Zn, Pb), 50 μM (Cu)	24 h; root	Antioxidant substances; antioxidant enzymes; transporters; transcription factors; cell wall components	Multi-heavy metal tolerance genes	Functional validation not reported [221]

More specifically, Cu stress in *S. alfredii* was associated with increased expression of HSFB2A, WRKY75, and ERF1B, together with changes in the metal transporters HMA5 and NRAMP3 and metal-homeostasis-related genes VIT1 and ALMT9. The same analysis identified the Ca^2+^ signaling proteins CML19 and CDPK34/17, suggesting an interconnected regulatory network involving transcription factors, Ca^2+^ signaling, and metal transport [221]. In *Z. mays*, Pb exposure altered transcription factors from the C2H2, bHLH, bZIP, AP2/ERF, and GARP families, further supporting the involvement of multiple TF classes in Pb-responsive transcriptional regulation [209].

Overall, transcriptomic studies demonstrate that plant responses to heavy metals are governed by interconnected molecular modules involving metal transport and sequestration, antioxidant and glutathione metabolism, hormonal and MAPK signaling, metabolic reprogramming, cell-wall remodeling, and transcriptional regulation.

## 6. Multi-Omics Approaches

### 6.1. Genomics

**Figure 3:**
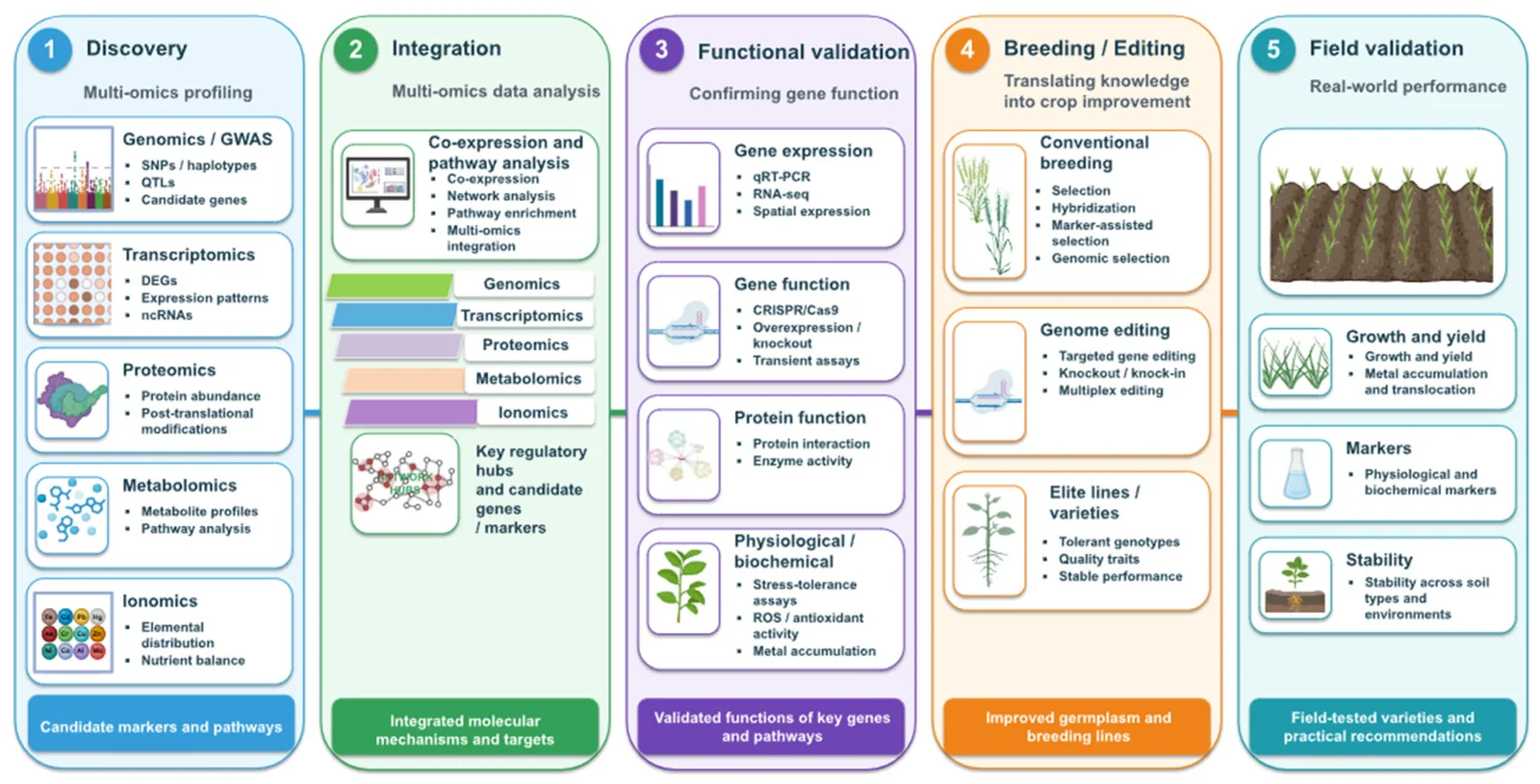
Integrated workflow for translating multi-omics discoveries into validated strategies for improving heavy-metal tolerance in crops. The framework follows a sequential workflow from discovery of candidate loci, genes, transcripts, proteins, metabolites, and elemental traits, through multi-omics integration and candidate prioritization, to functional validation using genetic, molecular, and biochemical approaches. Validated targets can then be applied in breeding and genome editing, followed by field validation to assess stability across environments, agronomic performance, metal accumulation, and essential-element homeostasis. The numbers (1–5) indicate the sequential stages of the proposed workflow, while different colors distinguish the five major stages. The icons are illustrative and represent the corresponding analytical methods and biological processes. Abbreviations: GWAS, genome-wide association study; SNP, single nucleotide polymorphism; QTL, quantitative trait locus; DEGs, differentially expressed genes; ncRNAs, non-coding RNAs: qRT-PCR, quantitative reverse transcription PCR; RNA-seq, RNA sequencing; CRISPR/Cas9, Clustered Regularly Interspaced Short Palindromic Repeats/CRISPR-associated protein 9. The figure was created using BioRender.com.

Genomic approaches provide a framework for linking natural genetic variation with differences in heavy-metal uptake, transport, accumulation, and detoxification (Figure 3). GWAS, QTL mapping, whole-genome sequencing (WGS), TILLING, comparative genomics, and CRISPR/Cas9-based functional genomics can be used at different stages of this process [20,231,232,233,234,235,236]. GWAS and QTL mapping analyses in wheat, for example, have identified loci associated with Cd uptake, translocation, and accumulation and have highlighted candidate transporter genes belonging to families such as NRAMP, ABC, and CCX [237,238,239,240,241,242,243]. approaches are particularly valuable because they connect measurable variation in metal accumulation with specific genomic regions rather than relying solely on stress-responsive gene expression.

However, identification of a QTL, SNP, or differentially responsive gene does not by itself establish its contribution to heavy-metal tolerance. Candidate-gene prioritization provides evidence of association between genetic variation and the phenotype, whereas functional genomics is required to determine whether a candidate directly contributes to metal accumulation, distribution, detoxification, or tolerance. For example, studies involving *TaEXPA2* and *TaCNR2* have provided functional evidence linking these genes with heavy-metal responses and altered heavy metal transport [244,245]. Similarly, comparative genomics analyses have identified candidate genes associated with phytochelatin biosynthesis and other detoxification processes [200,246], while regulatory studies have connected miRNAs with Cd-responsive transport genes such as *TaHMA2* [247]. These findings illustrate a progression from genomic association toward mechanistic interpretation, but the strength of evidence differs depending on whether candidates have been genetically manipulated and phenotypically validated.

An important distinction is therefore between genomic markers that predict variation in metal accumulation and genes that causally determine tolerance or elemental distribution. QTLs and SNPs can support marker-assisted or genomic selection even when the underlying causal gene has not been fully resolved, provided that the associated phenotype is reproducible across populations and environments [241,242,243]. In contrast, genetic modification, CRISPR/Cas-mediated perturbation, complementation, and corresponding physiological and elemental phenotyping provide stronger evidence for a direct functional role. From a crop-improvement perspective, genomic selection should therefore consider multiple traits simultaneously, including metal uptake and translocation, accumulation in edible tissues, essential-element homeostasis, growth, and yield. Thus, genomics is most informative when locus discovery is followed by functional validation and when genomic information can be translated into stable agronomic and food-safety traits in breeding populations.

### 6.2. Epigenomics

Epigenetic regulation represents an additional layer through which heavy-metal exposure can modify gene activity without altering the underlying DNA sequence. Changes in DNA methylation, histone modifications, and the profile of non-coding RNAs have been reported under Cd, Pb, Hg, Al, and As stress and are frequently associated with transcriptional reprogramming and stress acclimation [248,249,250,251]. The mechanistic significance of these changes, however, depends on whether an epigenetic modification precedes and regulates the corresponding transcriptional response or instead represents a downstream consequence of metal-induced stress.

DNA methylation has been associated with both genome protection and regulation of stress-response genes [252,253,254]. Heavy-metal-tolerant plants such as *C. sativa* and *Noccaea caerulescens* have been reported to show higher methylation levels and altered expression of methylation- and chromatin- related genes [252,255]. In rice, Cd exposure induced methylation changes that were associated with differential expression of genes involved in metal transport, metabolism, and transcriptional regulation [189,256]. These observations provide evidence for an association between epigenomic and transcriptional responses, but they do not necessarily show that the methylation changes drive the observed tolerance phenotype.

A stronger causal interpretation requires experimental manipulation of the epigenetic state, followed by assessment of gene expression and metal-related phenotypes. This distinction is particularly important when discussing stress memory and transgenerational inheritance. In *O. sativa*, heavy-metal exposure has been associated with CHG hypomethylation that persisted in subsequent generations and with increased resistance in offspring [257]. Similarly, methylation changes involving the Tos17 retrotransposon have been reported to persist across three generations [258]. These findings support the possibility that epigenetic modifications can contribute to heritable stress responses; however, persistence of an epigenetic mark alongside an altered phenotype does not, by itself, establish that the epigenetic modification is the causal determinant of transgenerational tolerance.

Thus, epigenomics provides important evidence for regulatory plasticity and potential stress memory, but its interpretation should distinguish between correlated epigenetic changes and experimentally demonstrated epigenetic control of heavy-metal tolerance. Integration with transcriptomic data, chromatin accessibility, targeted epigenome editing, genetic perturbation, and multigenerational phenotyping will be important for resolving this distinction.

### 6.3. Proteomics

Proteomics complements transcriptomic analysis by determining whether changes at the RNA level are reflected at the protein level [259,260,261]. This distinction is important because transcript abundance does not necessarily predict protein abundance, stability, localization, or activity. Proteomic studies of heavy-metal stress have therefore identified changes in proteins associated with ROS detoxification, redox regulation, molecular chaperoning, photosynthesis, respiration, and nitrogen and sulfur metabolism [261,262,263,264].

Across different metal stresses, several functional patterns occur. Proteins involved in antioxidant protection and redox homeostasis are frequently altered, whereas changes in photosynthetic and respiratory proteins indicate adjustment of energy metabolism and reducing-equivalent supply [259,262,263,264,265]. Alterations in Rubisco, oxygen-evolving complex proteins, NAD(P)H-dependent oxidoreductases, and photosystem I and II components illustrate the sensitivity of primary metabolism to heavy-metal exposure [265]. However, these changes should not automatically be interpreted as adaptive mechanisms. A decrease in photosynthetic proteins, for example, may represent either an active adjustment that limits oxidative damage or secondary damage to the photosynthetic apparatus.

The mechanistic value of proteomics therefore increases when protein abundance is interpreted together with enzymatic activity, metabolite levels, ROS measurements, elemental accumulation, and physiological phenotypes. Integration with metabolomics can connect changes in antioxidant- and stress-related proteins with proline, histidine, organic acids, glutathione, PCs, MTs, and ferritins, thereby providing a more coherent biochemical interpretation [259,260]. Nevertheless, even concordant protein accumulation does not establish causality unless the candidate protein’s function is experimentally perturbed and the resulting metal-related phenotype is assessed.

Thus, proteomics provides an important intermediate layer between transcriptional regulation and cellular phenotype, but its strongest contribution arises when protein abundance is linked to function and physiological outcomes rather than interpreted solely as an adaptive response to differential abundance.

### 6.4. Metabolomics

Metabolomics provides a biochemical layer that can reveal how heavy-metal exposure alters carbon, nitrogen, sulfur, lipid, redox, and secondary metabolism [260,266,267,268,269]. In contrast to genomic and transcriptomic approaches, which primarily identify potential regulatory changes, metabolomics measures downstream biochemical states and can therefore help determine whether molecular responses are accompanied by functional metabolic reprogramming.

Across different heavy metals and plant systems, several metabolic responses recur, although their direction and magnitude are strongly context dependent. Alterations in amino acids, carbohydrates, organic acids, lipids, phenolics, flavonoids, and phytohormone-associated metabolites have been reported under As, Al, Cu, Cd, and other metal stresses [270,271,272,273,274,275,276,277,278,279]. Rather than indicating a universal metal-specific metabolic signature, these changes point to several partially convergent processes, including maintenance of redox balance, adjustment of carbon and nitrogen metabolism, modulation of organic-acid pools, and reinforcement of secondary antioxidant metabolism. For example, changes in phenolics and amino acids under As and Al exposure have been associated with stress-related metabolic reprogramming, whereas accumulation of amino acids and flavonoids under Cd exposure has been linked with antioxidant responses [270,271,272,273,274]. Such observations illustrate common functional themes despite differences among species and metals.

Thiol metabolism provides a particularly informative connection between metabolomic responses and established detoxification mechanisms. Changes in glutathione and related metabolites under Cd and other metal stresses can be interpreted together with phytochelatin accumulation and glutathione-dependent detoxification pathways [275,276,277]. This integration provides stronger mechanistic evidence than changes in individual metabolites considered in isolation because it connects metabolic state with a defined biochemical pathway. Nevertheless, metabolite accumulation alone does not establish whether a metabolite actively mediates tolerance or accumulates as a consequence of stress.

Metabolomics is therefore most informative when metabolic changes are integrated with physiological and molecular measurements. For example, increased glutathione levels, enhanced phytochelatin production, altered expression of biosynthetic genes, reduced metal accumulation in sensitive cellular compartments, and improved physiological performance would provide substantially stronger evidence for a functional detoxification mechanism than any single metabolomic observation. Similarly, changes in phenolics, flavonoids, amino acids, or organic acids should be interpreted in relation to ROS status, antioxidant enzyme activity, metal distribution, and growth-related phenotypes rather than being automatically classified as adaptive responses.

Thus, metabolomics provides an important biochemical dimension to heavy-metal research, but its principal strength lies in linking metabolic signatures with upstream regulatory processes and downstream phenotypes. Importantly, a metabolite that changes in abundance under metal stress should not necessarily be interpreted as a mediator of tolerance. Demonstrating a functional contribution requires integration with complementary molecular, physiological, and, where possible, experimental validation. Distinguishing metabolic association from functional contribution is therefore essential when using metabolomic data to infer mechanisms of heavy-metal tolerance.

### 6.5. Ionomics

Ionomics provides a quantitative view of the elemental phenotype by profiling the distribution and accumulation of essential and nonessential elements in plant tissues. This approach is particularly useful for distinguishing changes in the target toxic metal from broader disturbances in nutrient homeostasis. For example, Cd exposure can modify the accumulation and translocation of Fe, Zn, Mn, and Cu, with the magnitude and direction of these interactions depending on plant species, tissue, exposure concentration, and element status [22].

The major advantage of ionomics is therefore its ability to reveal the systemic consequences of altered metal transport and homeostasis. However, elemental correlations alone do not identify the molecular mechanism responsible for the observed phenotype. A decrease in Fe or Zn under Cd exposure, for example, may result from competition for transport systems, altered transporter expression, changes in root-to-shoot translocation, or secondary disruption of nutrient metabolism. Consequently, ionomic patterns should be interpreted together with transporter expression, genetic variation, and functional experiments.

Integration with GWAS and QTL mapping provides a route from elemental phenotype to candidate genetic determinants. Loci and transporter genes associated with Cd accumulation [237,238,239,240,241,242,243] can be evaluated alongside tissue-specific elemental profiles to determine whether candidate variants explain differences in Cd accumulation and associated nutrient disturbances. Stronger mechanistic evidence is obtained when such associations are further supported by genetic perturbation and corresponding changes in elemental distribution and plant performance.

Thus, ionomics is particularly valuable for establishing the elemental consequences of heavy-metal exposure, whereas its ability to establish causality depends on integration with genomic, molecular, and functional evidence. In crop improvement, ionomic profiles can identify genotypes that restrict toxic-metal accumulation without disrupting essential-element homeostasis. Such combined phenotyping is particularly important because reducing toxic-metal transport may inadvertently disrupt Fe, Zn, Mn, or Cu homeostasis. In food crops, ionomic evaluation should therefore include metal concentrations in edible tissues alongside growth, yield, and essential-element traits, ensuring reduced toxic-metal accumulation without compromising plant performance or nutritional quality.

### 6.6. Integration of Multi-Omics Approaches

The main value of multi-omics analysis lies not in describing molecular datasets in parallel, but in integrating them to connect genetic variation with regulatory changes, biochemical states, elemental phenotypes, and plant performance. Each omics layer provides a distinct level of information: genomics identifies genetic variation and candidate loci; transcriptomics reveals responsive genes; epigenomics provides information on chromatin- and methylation-associated regulation; proteomics assesses protein-level changes; metabolomics captures biochemical states; and ionomics characterizes elemental composition, nutrient balance, and metal accumulation [19,21,280,281,282,283,284,285,286,287].

A useful framework organizes multi-omics studies as a progression from discovery to integration, functional validation, and phenotypic translation. GWAS, QTL mapping, transcriptomics, and related approaches can identify candidate loci, genes, pathways, and molecular signatures associated with heavy-metal responses. Concordant evidence across omics layers can then help prioritize candidates. For example, a genomic locus associated with Cd accumulation becomes more informative when accompanied by altered transporter expression, protein abundance, and corresponding elemental phenotypes. Similarly, coordinated changes in glutathione-related transcripts and proteins together with increased glutathione and phytochelatin levels provide convergent biochemical evidence [189,256,270,271,272,273,274,275,276,277,278,279].

Functional validation is required to determine whether prioritized candidates contribute directly to heavy-metal accumulation, detoxification, or tolerance. CRISPR/Cas-mediated perturbation, knockout or overexpression, complementation, promoter-reporter assays, molecular interaction studies, and targeted biochemical perturbation can be combined with quantitative physiological, molecular, and elemental phenotyping. Such experiments can distinguish candidate determinants from molecular responses that merely accompany metal-induced stress.

Validated targets can subsequently support breeding and biotechnology. Markers associated with reproducible elemental phenotypes may facilitate marker-assisted or genomic selection, whereas functionally validated genes may provide targets for genome editing or other molecular breeding strategies. Candidate loci and genes should nevertheless be evaluated across multiple traits, including metal uptake and translocation, tissue distribution, growth, yield, and essential-element homeostasis. Physiological tolerance alone does not necessarily indicate reduced metal accumulation and may, in some cases, coincide with increased retention in edible tissues.

Moreover, mechanisms identified under controlled conditions require validation across diverse environments because genotype × environment interactions, soil properties, nutrient availability, microbial associations, developmental stage, and metal exposure can modify the phenotype. For food crops, field validation should additionally assess metal concentrations in edible tissues and essential-element composition. Thus, future studies should prioritize hypothesis-driven multi-omics integration, functional validation, high-throughput phenotyping, and spatiotemporal modeling to distinguish reproducible biomarkers from functionally relevant determinants and translate validated mechanisms into stable agronomic traits [260].

The reliability of multi-omics data should be evaluated within the context of the biological question at hand. Consistency across multiple omics levels can increase confidence in a proposed mechanism but does not, in itself, establish a causal link. More compelling evidence is obtained when molecular changes are associated with reproducible physiological or elemental phenotypes and subsequently verified through targeted functional experiments. Heavy metal exposure simultaneously triggers stress responses, cellular damage, compensatory processes, and genuine adaptive mechanisms. Multi-omics datasets reflect these processes collectively, making it difficult to distinguish cause from effect based solely on observational data. Accordingly, data interpretation should proceed in a stepwise manner: from association to multi-omics integration, quantitative phenotyping, functional validation, and independent validation. This framework helps differentiate reproducible biomarkers from functionally significant determinants and supports experimentally grounded models of heavy metal tolerance.

## 7. Challenges and Future Perspectives

Despite significant progress in understanding plant tolerance to heavy metals, the strength and comparability of available evidence remain limited by differences in metal concentrations, exposure duration, tissue- and genotype-specificity, and experimental conditions. Several mechanisms, including metal uptake and transport, chelation and sequestration, ROS detoxification, and maintenance of cellular redox homeostasis, are relatively well established across multiple plant systems. However, the relative contribution of these mechanisms can vary substantially depending on the metal, plant species, genotype, tissue, developmental stage, and stress intensity. Therefore, mechanisms consistently observed across independent experimental systems should be distinguished from responses identified primarily through associative omics analyses.

A major challenge concerns the transition from association to functional evidence. Transcriptomic, proteomic, metabolomic, and epigenomic studies have identified numerous candidate genes, regulatory factors, proteins, and metabolic pathways associated with heavy-metal tolerance. However, changes in transcript abundance do not necessarily correspond to changes in protein abundance or activity, and many candidate genes identified by omics approaches have not been experimentally validated. Similarly, correlations between metabolite accumulation and tolerance do not necessarily establish a causal role in resistance. The spatial and temporal dynamics of these responses, their conservation across plant species, and their reproducibility under field conditions remain insufficiently characterized. The contribution of plant–microbiome interactions represents another promising but still incompletely resolved component of heavy-metal tolerance.

Future research should therefore prioritize the integration of multi-omics datasets with functional and phenotypic evidence. Single-cell and spatial transcriptomics, proteomics, metabolomics, and epigenomics can help resolve the cell-, tissue-, and time-specific organization of heavy-metal responses and identify regulatory networks that cannot be inferred from bulk analyses alone [19,21,288]. Integration of plant multi-omics data with metagenomic and metabolomic profiles of rhizosphere and endophytic microbiomes may further identify microorganisms, genes, and metabolic pathways associated with plant tolerance [238,289,290,291,292]. Such approaches should be combined with controlled experiments and field validation to determine whether identified molecular signatures are robust predictors of tolerance rather than context-dependent associations.

Among the most important priorities is the functional validation of candidate transporters, regulatory genes, and metabolic components identified through integrated omics analyses. Genomic regions and candidate genes associated with heavy-metal accumulation can provide targets for validation, while transcriptomic, proteomic, metabolomic, and ionomic data can be used to determine whether their effects are reflected at downstream molecular and physiological levels. CRISPR/Cas-based approaches are particularly valuable for testing causal relationships between candidate genes and heavy-metal uptake, transport, sequestration, and tolerance [21,286,287,288]. High-priority targets should therefore be selected not solely on the basis of differential expression, but according to the convergence of evidence across multiple omics layers and their association with measurable tolerance or metal-accumulation phenotypes.

AI and machine learning (ML) provide an additional tool to integrate multi-omic, phenotypic, and environmental datasets and identify bioinformative and predictive models of heavy-metal tolerance [21,287]. Combining deep learning with high-throughput phenomics and remote monitoring may improve early stress diagnosis and prediction of heavy-metal accumulation under various environmental conditions [21,287]. However, predictive performance should be evaluated using independent genotypes, environments, and field datasets to ensure that identified biomarkers are transferable beyond the experimental systems in which they were developed.

For crop improvement, the ultimate objective should be to translate robust multi-omic evidence into validated molecular markers, genomic selection strategies, and functionally supported targets for breeding and genome editing [283]. Multigene editing may provide opportunities to modify several components of heavy metals, while maintaining essential micronutrient homeostasis. Nevertheless, practical requires careful evaluation of off-target effects, environmental safety, as well as regulatory and ethical aspects [21,293,294].

Thus, future research should move from descriptive identification of stress-responsive features toward evidence-based prioritization of causal mechanisms and targets. The most reliable candidates will be those supported by convergent evidence across omics layers, reproducible across genotypes and environments, and confirmed by functional experiments. This hierarchy of evidence will be essential for distinguishing established mechanisms from promising associations and for translating multi-omics discoveries into effective crop improvement strategies.

## 8. Conclusions

Plant tolerance to heavy metals is a coordinated, multilevel process involving metal uptake and transport, chelation and sequestration, redox homeostasis, antioxidant defense, detoxification, and metabolic adaptation. These mechanisms are supported by substantial experimental evidence and therefore represent the most established basis for understanding plant tolerance. In contrast, many genes, transcription factors, proteins, metabolites, and epigenetic changes identified by multi-omics approaches remain primarily associative, with their functional significance depending on plant species, genotype, tissue, metal type, and exposure conditions.

Further progress will require the integration of multi-omic data with phenotypic and spatiotemporal information, followed by functional validation of candidate transporters, regulatory genes, and metabolic components. CRISPR/Cas-based approaches and AI-assisted analysis can facilitate the identification and validation of causal targets. Importantly, validated mechanisms should then be translated into reliable molecular markers, genomic selection strategies, and targeted breeding or genome-editing approaches to develop crops with enhanced heavy-metal tolerance and reduced metal accumulation.

Overall, the translation of multi-omics discoveries into effective plant breeding and biotechnology should follow a clear progression: established mechanisms, promising associations, functionally validated targets, and crop translation. This hierarchy provides a robust framework for distinguishing well-supported tolerance mechanisms from candidate molecular associations and for converting validated discoveries into practical strategies for crop improvement.

## Data Availability

No new data were created or analyzed in this study. Data sharing is not applicable to this article.

## Supplementary Materials

The following supporting information can be downloaded at: https://www.mdpi.com/article/10.3390/plants15192977/s1, Table S1: Literature search strategy used to identify relevant publications; Table S2: Transcriptomic responses, molecular pathways, candidate genes, and functional evidence associated with heavy-metal stress in plants.

## Abbreviations

The following abbreviations are used in this manuscript:

ChIP-seq	Chromatin immunoprecipitation sequencing
DAP-seq	DNA Affinity Purification Sequencing
