# Supplementary material for: Plant Responses to Heavy Metal Stress: Molecular Mechanisms, Transcriptomic Regulation, and Multi-Omics Integration in Crop Improvement
Source: Plants (Basel). 2026 Sep 29;15(19):2977. doi: 10.3390/plants15192977 (PMC13667007; doi:10.3390/plants15192977)
Supplement: Supplementary file 1 [file plants-15-02977-s001.zip › plants-4533514-supplementary.pdf]

Table S1. Literature search strategy used to identify relevant publications.

Database	Search strategy	Search period	Inclusion criteria	Exclusion Criteria
Web of Science Core Collection	TS = ((“heavy metal*” OR “metal stress” OR “metal toxicity”) AND (plant* OR crop*) AND (adaptation OR tolerance OR detoxification OR “metal homeostasis”) AND (“transcription factor*” OR transcriptom* OR “multi-omics” OR MAPK OR ROS OR “metal transport” OR chelation))	2010–September 2026	Peer-reviewed, English-language publications directly addressing plant responses to heavy-metal stress, including its molecular, physiological, biochemical, cellular, signaling, regulatory, detoxification, transport, and metal-homeostasis mechanisms, as well as studies employing transcriptomic, proteomic, metabolomic, ionomic, or other omics approaches	Dissertations and theses, conference abstracts or proceedings without sufficient peer-reviewed full-text information, newspaper and media reports, non-peer-reviewed technical reports, websites, blogs, opinion articles, and other non-scholarly sources, and studies that did not directly address plant responses to heavy-metal stress
Scopus	TITLE-ABS-KEY ((“heavy metal*” OR “metal stress” OR “metal toxicity”) AND (plant* OR crop*) AND (adaptation OR tolerance OR detoxification OR “metal homeostasis”) AND (“transcription factor*” OR transcriptom* OR “multi-omics” OR MAPK OR ROS OR “metal transport” OR chelation))	2010–September 2026	Same criteria	Same criteria
PubMed	((“heavy metal*”[Title/Abstract] OR “metal stress”[Title/Abstract] OR “metal toxicity”[Title/Abstract]) AND (plant*[Title/Abstract] OR crop*[Title/Abstract]) AND (adaptation[Title/Abstract] OR tolerance[Title/Abstract] OR detoxification[Title/Abstract] OR “metal homeostasis”[Title/Abstract]) AND (“transcription factor*”[Title/Abstract] OR transcriptom*[Title/Abstract] OR “multi-omics”[Title/Abstract] OR MAPK[Title/Abstract] OR ROS[Title/Abstract] OR “metal transport”[Title/Abstract] OR chelation[Title/Abstract]))	2010–September 2026	Same criteria	Same criteria

Table S2. Transcriptomic responses, molecular pathways, candidate genes, and functional evidence associated with heavy-metal stress in plants.

Plant species	Metal	Dose	Exposure duration, tissue	Key pathway	Candidate genes	Functional validation
V. radiata L.	Cd	100 μM	1, 5, and 9 days; root	Temporal transcriptional reprogramming; stress adaptation	NR*	Transcriptomic association only [176]
P. vaginatum	Cd	300 μmol/L	0, 1, 4 and 24 h; leaves	Metal transport; glutathione metabolism; transcriptional regulation	PvSnRK2.7	Functionally supported [177]
P.canadensis	Cd	10, 20 mg/L	3 months, leaves	Signal perception and transduction, transcription factors, antioxidant defense system and transporters	Potri.011G029800, Potri.011G029100, Potri.004G024140, Potri.011G030200, and Potri.004G023964	Transcriptomic association only [178]
A. donax L.	Cd	4 mg/kg)	3 months, leaves and roots	Protein biosynthesis; Phytohormone action; Nutrient uptake; Cell wall organisation; Polyamine metabolism; Reactive oxygen species metabolism; Ion membrane transport	NR	Transcriptomic association only [179]
C. indica	Cr	100 mg/kg	0, 7, 14, and 21 days	Carbohydrate metabolism; hormonal/MAPK signaling; glutathione and phenylpropanoid metabolism	NR	Transcriptomic association only [180]
C. sinensis	Cd, As	120 mg/kg	10, 15 days; roots	Stress signaling; transcriptional regulation	ERF; CSS0004428	Candidate association; validation not reported [181]
M. truncatula	Cd and Hg.	10 μM CdCl2 and 4 μM HgCl2	28 days; shoot, root, and nodules	Stress response; symbiosis; metal adaptation; detoxification mechanism; compartmentalization	NR	Transcriptomic association only [182]
P. americana	Cd	0, 50, 100, 150 or 200 mg/kg	5, 10 and 15 days; leaf, stem and root	Metabolism pathways; metal tolerance; absorption; transport and accumulation	NR	Transcriptomic association only [183]
G. hirsutum	Cd	0.5, 1, 2, 4, 6, 8 and 10 mM	1, 3, 6, 9, 12, 24 h; leaf, stem and root	Metal transport; redox response; tolerance mechanism	GhHMAD5	Functionally supported: GhHMAD5 contributed to Cd tolerance [184]

C. sativa	Cd	0, 2.5, 10, and 25 mg·L^{-1}	Root	Metal uptake; metal transport; sequestration	CsHMA1, CsHMA3, CsHMA4, CsHMA5	Transcriptomic association only [185]
S. orientalis	Cd	50 μM	1, 3, 5 days; shoot and root	ROS scavenging; phenylpropanoid biosynthesis pathway; Cd absorption and transport; and ABA signaling pathway	ABC transporter and YSL7	Transcriptomic association only [186]
Z. mays	Cd	5 mM	24 h; root	SAM metabolism; metal transport; vacuolar sequestration	ZmHMT3	Transcriptomic association only [188]
O. sativa	Cd	0.2, 1, or 50 *μ*M	14 days; root and shoot	Metal transport; Cd-responsive transcription	NR	Transcriptomic association only [189]
H. vulgare	Cd	80 *μ*M	5 days; shoot and root	Detoxification	HvPCR2	Candidate gene; functional validation not reported [190]
T. aestivum	Cd	0, 1, 3, and 10 mg/L	0, 2, 6, 12, 24, 48, and 72 h; root	Amino acid metabolism, ABC transporter activity, plant hormone signal transduction, phenylalanine metabolism, and flavonoid biosynthesis	YSL12; TraesCS2B02G394500; TraesCS2B02G394500	Candidate gene; functional validation not reported [191]
P. dactylifera L.	Cd	1.5 mg/kg^{-1}	20, 40, 60 days; leaves	Metal transport; chelation; detoxification	Pdpcs1, Pdmt3, Pdabcc, Pdhma2, Pdmate5, PdNramp6	Transcriptomic association only [192]
S. nigrum; S. torvum	Cd	50 *μ*M	12 h, 1, 3, 5 days; root	Metal transport; Fe homeostasis; antioxidant and amino-acid metabolism	NR	Transcriptomic association only [193]
H. macrophylla	Al	50 *μ*M	4h; leaves and root	Al tolerance; metal transport; Al uptake transporter, NRAMP aluminum transporter	NR	Transcriptomic association only [194]
F. esculentum	Al	0 or 20 μM	3 days; shoot and root	STOP/ART1-dependent Al tolerance; organic-acid transport	STOP1/ART1, FeSTAR1, FeALS3, FeALS1, FeMATE1 and FeMATE2	Candidate association; validation not reported [196]
F. tataricum	Al	0.5 mM	24h; root	ART1/STOP signaling; citrate transport; organic acid metabolism; antioxidant and detoxification pathways	STAR1, STAR2/ALS3, ALS1, FtFRDL1, FtFRDL2	Candidate association; validation not reported [195]
M. sativa	Pb	0 or 200 mg/L	0, 24, 48, 72, 96, or 120 h; root	Metal transport; antioxidant defense; flavonoid metabolism; secondary and glutathione metabolism;	MTs, antioxidant enzymes, secondary and glutathione	Transcriptomic association only [197]

				photosynthesis; signal sensing and transduction; transcriptional regulation	metabolism, photosynthesis, TF, signal sensing and transduction proteins candidate genes	
F. tataricum	Pb	0, 1000, 2000, 5000, or 10,000 mg/kg Pb	72h; leaves	Metal transport; phytochelatin-mediated detoxification; primarily associated with primary defence mechanisms; such as cell walls; plant hormone signal transduction; the antioxidant system and photosynthesis.	ZFP14, MTPC2, PCSL, VCE1a, Nramp3, PCs genes	Transcriptomic association only [198]
L. chuanxiong	Cd	0, 5, 10, 20, 40 mg/L^{-1}		Hormonal signaling; antioxidant defense; cell-wall modification; transport/sequestration	MT genes	Transcriptomic/physiological association; validation not reported [199]
T. aestivum L.	Cd	15 μM	7 days; root and shoot	Hormonal signaling; antioxidant defense; cell-wall	NR	Transcriptomic association only [200]
B. juncea L.	Mn, Fe, Zn, Cd, Sb and Pb	250 mg Mn^{2+}, 250 mg Fe^{2+}, 200 mg Zn^{2+}, 100 mg Cd^{2+}, 10 mg Sb^{3+}, and 250 mg Pb^{2+}	12, 24, and 48 h; leaves	Metal transport; metal-specific regulatory responses	BjMTP genes	Transcriptomic association only [201]
Populus x canadensis	Zn	1 μM; 1 mM	21 days; leaves	Fe transport; cellular homeostasis; chelation and detoxification	ZIP2, NRAMP1, FIT, BTS	Transcriptomic association only [202]
C. sinensis	Cd, Al, Cu	5.6-53.4 (Cd), 7349.6-7843.3 (Al), 32.3-33.3 mg/kg (Cu)	Natural condition; leaves and root	Metal transport; cultivar-specific metal accumulation	MT genes	Transcriptomic association only [203]

A. thaliana	Cd, Cu	10, 50 μM Cd), 10 μM (Cu)	10-12 days; root	Metal-specific signaling; Zn deficiency; Cd hyper-tolerance	Candidate Cd-tolerance genes	Candidate association; validation not reported [204]
A. halleri	Cd, Cu	25, 125 μM Cd), 10 μM (Cu)	10-12 days; root	Metal-specific signaling; Zn deficiency; Cd hyper-tolerance	Candidate Cd-tolerance genes	Candidate association; validation not reported [204]
H. vulgare	Cd	20 μmol L^{-1}	24h; root	lncRNA–mRNA regulation; ABC transport; detoxification	26 lncRNAs; 150 mRNAs	Regulatory association; functional validation not reported [205]
S. aculeatissimum	Cd	3, 10, 20, 30, 40, 50 40 mg/kg^{-1}	90, 163 days; stem, leaves, and root	HMA-mediated transport; phytochelatin; antioxidant defense	SaHMA20, SaL-AO, SaPrxs4, SaPCs	Functionally associated with Cd detoxification/hyperaccumulation [206]
Gossypium spp.	Cd	0, 1, 5, 10 mmol/L	10 days, root	Antioxidant defense; flavonoid biosynthesis; metal transport	NR	Transcriptomic association only [207]
H. vulgare	Cd	5, 50 and 500 μM	15 days; leaves	Carbohydrate metabolism; antioxidant defense; ABA/JA responses; transport	Cd-responsive genes	Transcriptomic association only [208]
Z. mays	Pb	1,000 mg/L^{-1}	6, 12, 24, 48, 72, 96h	Antioxidant defense; transcriptional regulation	NR	Transcriptomic association only [209]
A. hypogaea	Cd	2.5 mg/kg	15 days, leaves	Antioxidant defense; cell wall biosynthesis; secondary metabolism; plant hormone signal transduction	AUX/IAA, GH3, SAUR, JAZ	Physiological/transcriptomic evidence; causal validation not reported [210]
Z. mays	Cd	20 mM	72h; stems, leaves and root	Tissue-specific response patterns; TF	NR	Transcriptomic association only [232]
P. crinitum	Pb	0, 300, 500, 1000, 2000 mg·L^{-1}	7 days; root	Antioxidant defense; metal transport; MAPK pathway; transcriptional regulation	17 Pb-tolerance-associated genes	Transcriptomic association only [211]
Brassica rapa	Cd and Pb	80 (Cd), 1200 (Pb) mg/L	10 days; root	Antioxidant defense; MAPK/calmodulin signaling; phenylpropanoid metabolism	ARR-B, AP2-EREBP, NAC, bHLH, WRKY, and bZIP genes	Transcriptomic association only [212]
A. thaliana	As	100 to 300 μM	2 days; root	Oxidative/heat stress; ethylene signaling	LRR-RLK VIII; HSPs; HSFs	Candidate LRR-RLK VIII associated with As tolerance;

						functional validation not reported [213]
O. sativa	Cd, As	50 (Cd) and 25 (As) µM	3 (Cd) and 7 (As) days; root	Redox control; stress response; transcriptional regulation; transmembrane transport; signal transduction; macromolecules biosynthesis	NR	Transcriptomic association only [214]
B. nigra	Cu	500 µM	NR; root	Glutathione metabolism; metal transport	NR	Transcriptomic association only [215]
H. vulgare	Co, Cu	50 µM	1, 2, 5 and 10 days; leaves	MAPK signaling, glutathione; phenylalanine; anthocyanin/flavonoid metabolism	NR	Transcriptomic association only [216]
P. banksiana	Ni	1600 mg/kg	14 days; leaves	Stress response; carbohydrate metabolism; catabolic processes	NR	Transcriptomic association only [222]
O. sativa	Cd, As, Pb, Cr	0, 25, 50, 100 and 200 µM	7 days; root	Secondary metabolism; GST; ABC/MFS transport; MAPK signaling	NR	Transcriptomic association only [223]
Z. mays	Al	10 $mmol_c/dm^3$	5 days; leaves and root	The tricarboxylic acid (TCA) cycle; auxin; GA; JA; brassinosteroid signaling	Hormone-responsive genes	Transcriptomic association only [220]
F. arundinacea	Pb	1,000 mg L^{-1}	4, 48h; leaves	Energy metabolism; terpenoids and polyketides metabolism; carbohydrate metabolism	NR	Transcriptomic association only [224]
Brassica spp.	Cd	5 mg L^{-1}	7 days; root	Auxin-affected cadmium accumulation	NR	Transcriptomic association only [217]
H. cannabinus	Cd	10 mg L^{-1}	7 days; leaves	Activation of antioxidant enzymes, MAPK signaling, and detoxification	NR	Transcriptomic association only [218]
S. babylonica	Co	50, 100, 200, 400 µM	30 days; stem, leaves and root	Antioxidant defense; phytohormone signaling; oxidative stress; TFs, and detoxification	NR	Transcriptomic association only [219]
C. lanceolata	Al	1 mmol/L	1, 72h; root	Cell wall and lipid metabolism; signaling pathways; secondary metabolism involving flavonoids and phenylpropanoids; TF	NR	Transcriptomic association only [225]
V. bonariensis	Cd	100 mg/L	20 days; root	ROS scavenging system; chelating reaction; secondary metabolites, transpiration and photosynthesis.	NR	Transcriptomic association only [226]

G. max	Al	25 μM	6, 12 or 24 h; root	Cellular glucan metabolic process; regulation of transcription	Glyma.02G205800	Functional validation not reported [227]
G. max	Cd	75 lmol·L^{-1}	7 days; root	Antioxidants; sequestration; metal-transporting	GmHMA5	Functional validation [228]
G. max	Cd	50 μM	72h; root	Plant-pathogen interaction; Phenylpropanoid biosynthesis; Phenylalanine metabolism; Carbon metabolism; Starch and sucrose metabolism; Glutathione metabolism; Protein processing in endoplasmic reticulum pathways	NR	Transcriptomic association only [229]
Sedum alfredii	Cd	5, 100 mM	8 days; shoot and root	cell wall modification; vacuolar sequestration in; metal translocation; remobilization	NR	Transcriptomic association only [230]
S. alfredii	Cd, Zn, Pb, Cu	100 μM (Cd, Zn, Pb), 50 μM (Cu)	24h; root	Antioxidant substances; antioxidant enzymes; transporters; transcription factors; cell wall components	Multi-heavy metal tolerance genes	Functional validation not reported [221]
G. hirsutum	Cd	200 μmol L^{-1}	7 days; leaves	Detoxification; antioxidant enzymes; valine, leucine and isoleucine degradation; ABC transporter; alpha-linolenic acid metabolism; flavonoids and alkaloids; accumulation of secondary metabolites; chelation, such as amino acids and derivatives; ion transportation; ABC transporter activation	NR	Transcriptomic association only [231]

Note: * NR – not reported
